# Supplementary material for: Diaryl Bismuthides and Acyl Bismuthanes Enable Visible-Light-Induced Reversible Carbon Monoxide Insertion and Extrusion
Source: Nat Commun. 2025 Nov 20;16:10234. doi: 10.1038/s41467-025-66412-5 (PMC12635283; doi:10.1038/s41467-025-66412-5)
Supplement: Supplementary file 1 — Supplementary Information [file 41467_2025_66412_MOESM1_ESM.pdf]

Supplementary Information

Diaryl Bismuthides and Acyl Bismuthanes Enable Visible-Light-Induced Reversible Carbon Monoxide Insertion and Extrusion

Felix Geist,[1] Benedikt Narz,[1] Darian Boguslawski,[1] Tobias Dunaj,[1] Sascha Reith,[1] Jordi Poater,[2,3] and Crispin Lichtenberg[1,*]

1 F. Geist, B. Narz, D. Boguslawski, Dr. T. Dunaj, S. Reith, Prof. Dr. C. Lichtenberg
Department of Chemistry
Philipps-Universität Marburg
Hans-Meerwein-Str. 4, 35032 Marburg, Germany
E-mail: crispin.lichtenberg@chemie.uni-marburg.de

2 Prof. Dr. J. Poater
Departament de Química Inorgànica i Orgànica & IQTCUB
Universitat de Barcelona, 08028 Barcelona, Spain

3 ICREA, 08010 Barcelona, Spain

Table of contents

Experimental section S2

General considerations S2

Synthesis of dibismuthanes **1-4** S3

Synthesis of [K(crypt)][$C_{14}H_{10}Bi$] (**5**) S4

Synthesis of [K(crypt)][Mes_2Bi] (**6**) S5

Synthesis of [K(crypt)][Ph_2Bi] (**7**) S5

Synthesis of [K(crypt)][$Dipp_2Bi$] (**8**) S6

Synthesis of $C_{14}H_{10}BiC(=O)Ad$ (**9**) S7

Synthesis of $Mes_2BiC(=O)Ad$ (**10**) S7

Synthesis of $C_{14}H_{10}BiAd$ (**11**) S8

Synthesis of Mes_2BiAd (**12**) S9

Single-Crystal X-ray Diffraction Analysis S10

UV/Vis spectra S13

Thermochromism of diaryl bismuthides S16

Reactions with (^{13}C-labeled) carbon monoxide S18

Main group carbonyl and acyl species S18

Reactivity of **11** towards CO S20

Reactivity of **12** towards CO S22

Reactivity of **9** towards ^{13}CO S23

Reactivity of **10** towards ^{13}CO S26

IR spectra S28

NMR spectra S30

Mass spectra S39

Quantum chemical calculations S44

References S48

Experimental section

General considerations

All air- and moisture-sensitive manipulations were carried out using standard vacuum line Schlenk techniques or in gloveboxes containing an atmosphere of purified argon. Solvents were degassed and purified according to standard laboratory procedures. **NMR spectra** were recorded on Bruker instruments operating at 300, 400 or 500 MHz with respect to ^{1}H. ^{1}H and ^{13}C NMR chemical shifts are reported relative to $SiMe_4$ using the residual ^{1}H and ^{13}C chemical shifts of the solvent as a secondary standard. NMR spectra were recorded at ambient temperature (typically 20 °C), if not otherwise noted. **Elemental analyses** were performed on a Vario Micro Cube by Elementar Analysesysteme GmbH. **UV-vis** measurements were performed under an inert gas atmosphere in quartz glass cuvettes (10 mm) on an Analytik-Jena SPECORD S 600 spectrometer. **IR spectra** were recorded in pure substance on a Bruker Alpha II spectrometer. **Single-crystal X-ray diffraction data** were collected either on a STOE IPDS 2T diffractometer or on a Bruker D8-QUEST diffractometer using MoKα radiation (λ = 0.71073 Å). The respective solid-state structures were solved using intrinsic phase methods (ShelXT), refined with the ShelX software package and developed using Fourier techniques.[1,2] All non-hydrogen atoms were refined anisotropically. Hydrogen atoms were assigned to idealized positions. The crystallographic data used in the publication were deposited with the Cambridge Crystallographic Data Center (CCDC) and are available at https://www.ccdc.cam.ac.uk/structures. The images of the solid-state structures were created with the Mercury software. HR-ESI and HR-APCI **mass spectra** were acquired with an Orbitrap Q Exactive plus mass spectrometer (Thermo Fischer Scientific). The resolution was set to 140.000. HR-LIFDI mass spectra were acquired with an AccuTOF GCv 4G (JEOL) Time of Flight (TOF) mass spectrometer. An internal or external standard was used for drift time correction. The LIFDI ion source and FD-emitters were purchased from Linden ChroMasSpec GmbH (Bremen, Germany). **Photochemical experiments** were carried out in a MPDSBasic photonCABINET photoreactor from KARLBIEDEN, equipped with a mercury vapor lamp of the type LOT Quantum Design LSB740 (I = 19 A, U = 26 V, 400–500 W) with IR filters irradiating at 210–600 nm and photoLAB LED radiation sources of wavelengths 365 (Φe = 43.2 W) or 460 nm (Φe = 26.6–45.0 W). thermoCONTROL 100 functions as the cooling unit. All experiments were carried out using borosilicate glassware.

The following labeling scheme was used for reporting NMR chemical shifts of the dibenzobismepine species **5**, **9**, and **11**:

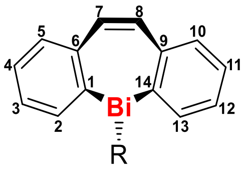

Synthesis of dibismuthanes **1-4**

The dibismuthanes $Bi_2(C_{14}H_{10})_2$ (**1**), Bi_2Ph_4 (**3**), and Bi_2Dipp_4 (**4**) were prepared according to (modified) literature procedures.[3–5] Bi_2Mes_4 (**2**) was prepared analogous to **4**.[4]

$Bi_2(C_{14}H_{10})_2$ (**1**): 5-Iodo-5*H*-dibenzo[*b*,*f*]bismepine (500 mg, 973 µmol) was dissolved in THF (8 mL) and a solution of cobaltocene (184 mg, 973 µmol) in THF (3 mL) was added. The reaction mixture was filtered, the filtrate layered with *n*-pentane (11 mL) and stored at −30 °C. After 16 h the product had precipitated as an orange solid which was isolated by filtration, washed with *n*-pentane (3×4 mL) and dried in vacuo. **Yield**: 355 mg (458 µmol, 94 %). **^{1}H NMR** (400 MHz, THF-d_8): δ = 6.59 (s, 4H, *H*-7, *H*-8), 7.04–7.12 (m, 8H, *H*-3, *H*-4, *H*-11, *H*-12), 7.19 (dd, 4H, $^3J_{HH}$ = 7.1 Hz, $^4J_{HH}$ = 1.8 Hz, *H*-5, *H*-10), 7.90 (dd, 4H, $^3J_{HH}$ = 6.6 Hz, $^4J_{HH}$ = 2.0 Hz, *H*-2, *H*-13) ppm.

Bi_2Mes_4 (**2**): A solution of $NaBHEt_3$ (1 M in toluene, 3.6 mL, 3.60 mmol) was added to a solution of dimesitylbismuth chloride (1.50 g, 3.11 mmol) in diethyl ether (50 mL) at −78 °C. The reaction mixture was allowed to warm to room temperature over two hours. After separation of a precipitate *via* filtration, all volatiles were evaporated under reduced pressure. The product was obtained as a dark brown solid. **Yield:** 888 mg (992 µmol, 64 %). **^{1}H NMR** (300 MHz, C_6D_6): 2.12 (s, 12H, C*H*$_3$-para), 2.39 (s, 24H, C*H*$_3$-ortho), 6.85 (s, 8H, Ar*H*) ppm.

Bi_2Ph_4 (**3**): A solution of cobaltocene (193 mg, 1.02 mmol) in THF (6 mL) was added to a solution of diphenylbismuth iodide (500 mg, 1.02 mmol) in THF (20 mL). The reaction mixture was filtered, and all volatiles were removed under reduced pressure. The residue was extracted with toluene (10 mL), the solution layered with *n*-pentane (10 mL) and stored at −30 °C. After 16 h the product had precipitated as an orange solid which was isolated by filtration, washed with *n*-pentane (3×2 mL) and dried in vacuo. **Yield:** 246 mg (339 µmol, 68 %). **^{1}H NMR** (300 MHz, C_6D_6): δ = 7.00−7.15 (m, 12H, Ar*H*-meta/para), 7.90 (d, 8H, $^3J_{HH}$ = 7.6 Hz, Ar*H*-ortho) ppm.

Bi_2Dipp_4 (**4**): A solution of $NaBHEt_3$ (1 M in toluene, 0.64 mL, 0.64 mmol) was added to a solution of $Dipp_2BiBr$ (400 mg, 0.64 mmol) in toluene (30 mL) at −78 °C. The reaction mixture

was allowed to warm to room temperature over four hours. After separation of a precipitate *via* filtration, all volatiles were evaporated under reduced pressure. The remaining residue was washed with acetonitrile and dibismuthane **4** obtained as a dark brown solid. **Yield:** 290 mg (273 mmol, 85%). **^{1}H NMR** (300 MHz, C_6D_6): δ = 0.89 (d, 24H, $^3J_{HH}$ = 6.5 Hz, CH(C*H*$_3$)$_2$), 1.14 (d, 24H, $^3J_{HH}$ = 6.5 Hz, CH(C*H*$_3$)$_2$), 3.23 (sept, 8H, $^3J_{HH}$ = 6.5 Hz, C*H*(CH$_3$)$_2$), 7.08−7.22 (m, 8H, Ar*H*) ppm.

Synthesis of [K(crypt)][$C_{14}H_{10}$Bi] (**5**)

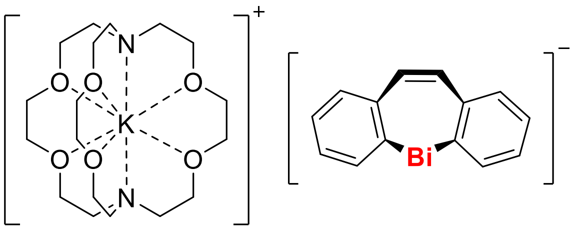

Potassium graphite (KC_8, 87 mg, 646 µmol) was added to a suspension of dibismuthane **1** (250 mg, 323 µmol) and [2.2.2]cryptand (243 mg, 646 µmol) in THF (10 mL). The reaction mixture was stirred at rt for 1 min and then filtered. The filtrate was layered with *n*-pentane (10 mL) and stored at −30 °C. After 1 d dark orange crystals of **5** had formed, which were isolated by filtration, washed with *n*-pentane (3 x 3 mL) and dried *in vacuo*. **Yield:** 436 mg, 543 µmol, 84%.

^{1}H NMR (500 MHz, THF-*d*$_8$): δ = 2.47 (t, 12H, crypt), 3.46 (t, 12H, crypt), 3.51 (s, 12, crypt), 6.22 (s, 2H, *H*7/8), 6.39 (t, 2H, $^3J_{HH}$ = 6.9 Hz, *H*4/11), 6.49 (t, 2H, $^3J_{HH}$ = 7.1 Hz, *H*3/12), 6.62 (d, 2H, $^3J_{HH}$ = 7.5 Hz, *H*5/10), 8.42 (d, 2H, $^3J_{HH}$ = 7.1 Hz, *H*2/13) ppm. **^{1}H NMR** (500 MHz, pyridine-*d*$_5$): δ = 2.37 (t, 12H, crypt), 3.37 (t, 12H, crypt), 3.42 (s, 12, crypt), 6.90 (s, 2H, *H*7/8), 6.93 (t, 2H, $^3J_{HH}$ = 7.1 Hz, *C*4/11), 6.99 (ddd, 2H, $^3J_{HH}$ = 7.3 Hz, $^4J_{HH}$ = 1.2 Hz, *C*3/12), 7.21 (d, 2H, $^3J_{HH}$ = 7.6 Hz, *C*5/10), 9.30 (d, 2H, $^3J_{HH}$ = 7.1 Hz, *H*2/13) ppm. **^{13}C{^{1}H} NMR** (126 MHz, THF-*d*$_8$): δ = 54.89 (s, crypt), 68.57 (s, crypt), 71.36 (s, crypt), 122.87 (s, *C*3/12), 128.98 (s, *C*4/11), 130.14 (s, *C*5/10), 142.29 (s, *C*7/8), 146.15 (s, *C*2/13), 152.6 (s, *C*6/9, detected via ^{13}C/^{1}H HMBC experiments) ppm. A resonance for *C*1/C14 could not be detected via regular ^{13}C NMR or ^{13}C/^{1}H HMBC experiments. **^{13}C{^{1}H} NMR** (126 MHz, pyridine-*d*$_5$): δ = 54.37 (s, crypt), 68.14 (s, crypt), 70.89 (s, crypt), 123.41 (s, *C*3/12), 130.03 (s, C4/11), 131.01 (s, *C*5/10), 143.12 (s, *C*7/8), 145.53 (s, *C*1/14), 146.80 (s, *C*2/13), 152.56 (s, *C*6/9) ppm. **HR-MS (ESI, neg.):** calculated for ($^{12}C_{14}{}^1H_{10}{}^{209}Bi)^-$: *m/z* = 387.0581, found: *m/z* = 387.0591. **UV-vis (THF):** λ_{max} = 357 nm, 430 nm, onset at 570 nm. **Elemental analysis:** Anal. calc. (%) for $C_{32}H_{46}BiKN_2O_6$ (802.81 g · mol^{-1}): C 47.88, H 5.78, N 3.49; found: C 47.94, H 6.13, N 3.53.

Synthesis of [K(crypt)][Mes_2Bi] (**6**)

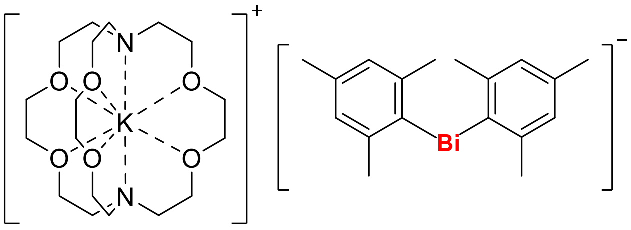

Potassium graphite (KC_8, 37 mg, 271 μmol) was added to a solution of tetramesityl dibismuthane (**2**, 121 mg, 135 μmol) and [2.2.2]cryptand (102 mg, 271 μmol) in THF (8 mL). The reaction mixture was stirred at rt for 1 min and then filtered. The filtrate was layered with *n*-pentane (10 mL) and stored at −30 °C. After 1 d red crystals of **6** had formed, which were isolated by filtration, washed with *n*-pentane (3 x 2 mL) and dried *in vacuo*. **Yield:** 147 mg, 170 μmol, 63%.

^{1}H NMR (500 MHz, THF-d_8): δ = 2.16 (s, 6H, CH_3-para), 2.31 (s, 12H, CH_3-ortho), 2.49 (t, 12H, crypt), 3.49 (t, 12H, crypt), 3.53 (s, 12H, crypt), 6.60 (s, 4H, Ar*H*) ppm. **^{13}C{^{1}H} NMR** (126 MHz, THF-d_8): δ = 20.82 (s, *C*H_3-para), 41.75 (s, *C*H_3-ortho), 54.85 (s, crypt), 68.53 (s, crypt), 71.32 (s, crypt), 123.38 (s, Ar*C*-meta), 130.70 (s, Ar*C*-meta), 146.94 (s, Ar*C*-ortho), 157.33 (s, Ar*C*-ipso) ppm. **HR-MS (ESI, neg.):** calculated for ($^{12}C_{18}{}^{1}H_{22}{}^{209}Bi)^-$: *m/z* = 447.1531, found: *m/z* = 447.1528. **UV-vis (THF):** λ_{max} = 222 nm, 297 nm, 383 nm, onset at 455 nm. **Elemental analysis:** Anal. calc. (%) for $C_{36}H_{58}BiKN_2O_6$ (862.95 g · mol^{-1}): C 50.11, H 6.77, N 3.25; found: C 50.33, H 7.10, N 3.38.

Synthesis of [K(crypt)][Ph_2Bi] (**7**)

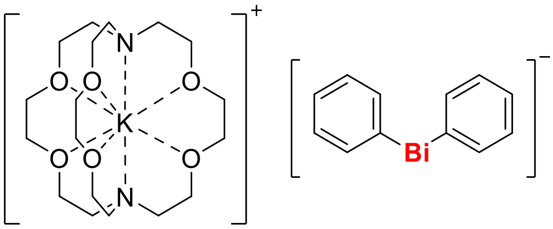

Potassium graphite (KC_8, 57 mg, 420 μmol) was added to a suspension of tetraphenyl dibismuthane (**3**, 150 mg, 210 μmol) and [2.2.2]cryptand (158 mg, 420 μmol) in THF (6 mL). The reaction mixture was stirred at rt for 1 min and then filtered. The filtrate was layered with *n*-pentane (6 mL) and stored at −30 °C. After 1 d red crystals of **7** had formed, which were isolated by filtration, washed with *n*-pentane (3 x 3 mL) and dried *in vacuo*. **Yield:** 125 mg, 543 μmol, 38%.

^{1}H NMR (500 MHz, THF-d_8): δ = 2.49 (t, 12H, crypt), 3.48 (t, 12H, crypt), 3.53 (s, 12H, crypt), 6.53 (t, 2H, $^3J_{HH}$ = 6.5 Hz, Ar*H*-para), 6.67 (t, 4H, $^3J_{HH}$ = 7.2 Hz, Ar*H*-meta), 8.20 (dd, 4H, $^3J_{HH}$

= 7.6 Hz, $^4J_{HH}$ = 1.2 Hz, Ar*H*-ortho) ppm. **^{13}C{^{1}H} NMR** (126 MHz, THF-d_8): δ = 54.91 (s, crypt), 68.59 (s, crypt), 71.37 (s, crypt), 122.06 (s, Ar*C*-para), 128.68 (s, Ar*C*-meta), 144.60 (s, Ar*C*-ortho), 150.47 (s, Ar*C*-ipso) ppm. **HR-MS (ESI, neg.):** calculated for $(^{12}C_{12}{}^{1}H_{10}{}^{209}Bi)^{-}$: *m/z* = 363.0592, found: *m/z* = 363.0597. **UV-vis (THF):** λ_{max} = 221 nm, 394 nm, 249 nm, 284 nm, 320 nm, onset at 560 nm. **Elemental analysis:** Anal. calc. (%) for $C_{30}H_{46}BiKN_2O_6$ (778.78 g · mol^{-1}): C 46.27, H 5.95, N 3.60; found: C 45.79, H 5.61, N 3.86.

Synthesis of [K(crypt)][Dipp$_2$Bi] (**8**)

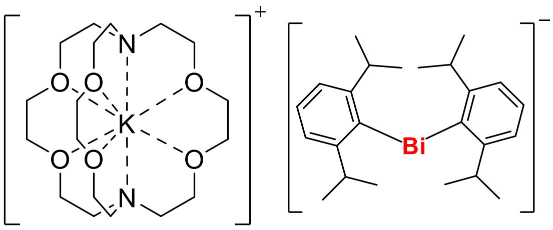

Potassium graphite (KC_8, 46 mg, 339 µmol) was added to a solution of dibismuthane **4** (180 mg, 169 µmol) and [2.2.2]cryptand (128 mg, 339 µmol) in THF (8 mL). The reaction mixture was stirred at rt for 1 min and then filtered. The filtrate was layered with *n*-hexane (6 mL) and stored at −30 °C. After 1 d black crystals of **8** had formed, which were isolated by filtration, washed with *n*-pentane (3 x 3 mL) and dried *in vacuo*. **Yield:** 211 mg, 223 µmol, 66%.

^{1}H NMR (500 MHz, THF-d_8): δ = 0.95 (d, 24H, $^3J_{HH}$ = 6.9 Hz, C*H*$_3$), 2.52 (s, 12H, crypt), 3.51 (t, 12H, crypt), 3.54 (s, 12H, crypt), 3.72 (sept, 4H, $^3J_{HH}$ = 6.9 Hz, C*H*), 6.58 (t, 2H, $^3J_{HH}$ = 7.5 Hz, Ar*H*-para), 6.85 (d, 4H, $^3J_{HH}$ = 7.5 Hz, Ar*H*-meta) ppm. **^{13}C{^{1}H} NMR** (126 MHz, THF-d_8): δ = 26.04 (s, *C*H$_3$), 54.86 (s, crypt), 56.68 (s, *C*H), 68.61 (s, crypt), 71.48 (s, crypt), 119.46 (s, Ar*C*-meta), 125.55 (s, Ar*C*-para), 157.78 (s, Ar*C*-ortho), 172.66 (s, Ar*C*-ipso) ppm. **HR-MS (ESI, neg.):** calculated for $(^{12}C_{24}{}^{1}H_{34}{}^{209}Bi)^{-}$: *m/z* = 531.2459, found: *m/z* = 531.2465. **UV-vis (THF):** λ_{max} = 222 nm , 394 nm, onset at 605 nm. **Elemental analysis:** Anal. calc. (%) for $C_{42}H_{70}BiKN_2O_6$ (947.11 g · mol^{-1}): C 53.26, H 7.45, N 2.96; found: C 53.42, H 7.38, N 3.03.

Synthesis of $C_{14}H_{10}BiC(=O)Ad$ (**9**)

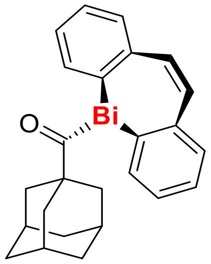

A solution of 1-adamantanecarbonyl chloride (99 mg, 498 µmol) in THF (4 mL) was added to a suspension of bismuthide **5** (400 mg, 498 µmol) in THF (10 mL). The yellow suspension was filtered and all volatiles removed under reduced pressure. The residue was extracted with a mixture of toluene (4 mL) and THF (10 mL), layered with *n*-pentane (8 mL) and stored at −30 °C. After 3 d yellow crystals of **9** had formed, which were isolated by filtration, washed with *n*-pentane (3 x 2 mL) and dried *in vacuo*. **Yield:** 170 mg, 309 µmol, 62%.

Due to the rapid decomposition of compound **9** in solution at rt NMR spectra were recorded at 273 K.

^{1}H NMR (500 MHz, CD_2Cl_2): δ = 1.64−1.80 (m, 6H, Ad-CH_2), 1.83 (d, 6H, $^3J_{HH}$ = 2.6 Hz, Ad-CH_2), 2.07−2.15 (m, 3H, Ad-C*H*), 6.77 (s, 2H, *H*7/8), 7.25−7.33 (m, 4H, *H*3/4/11/12), 7.42−7.46 (m, 2H, *H*5/10), 7.82−7.86 (m, 2H, *H*2/13) ppm. **^{13}C{^{1}H} NMR** (126 MHz, CD_2Cl_2): δ = 28.52 (s, Ad-*C*H), 36.71 (s, Ad-*C*H_2), 37.97 (s, Ad-*C*H_2), 60.38 (s, Ad-*C*), 127.63 (s, *C*3/12), 129.97 (s, *C*4/11), 131.05 (s, *C*5/10), 134.12 (s, *C*7/8), 137.64 (s, *C*2/13), 143.41 (s, *C*6/9), 155.56 (s, *C*1/14), 253.81 (s, C=O) ppm. **UV-vis (DCM):** λ_{max} = 250 nm, 281 nm, 320 nm, 400 nm, onset at 500 nm. **Elemental analysis:** Anal. calc. (%) for $C_{25}H_{25}BiO$ (550.45 g · mol^{-1}): C 54.55, H 4.58; found: C 54.85, H 4.44. **IR (neat):** $\tilde{\nu}_{CO}$ = 1691 (s) cm^{-1}.

Synthesis of $Mes_2BiC(=O)Ad$ (**10**)

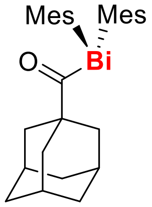

A solution of 1-adamantecarbonyl chloride (35 mg, 174 µmol) in THF (2 mL) was slowly added to a solution of bismuthide **6** (150 mg, 174 µmol) in THF (5 mL). The yellow suspension was filtered and all volatiles were removed under reduced pressure. The residue was extracted with *n*-pentane (3 x 2 mL) and stored at −30 °C. After 2 d orange crystals of **10** had formed, which were isolated by filtration and dried *in vacuo*. **Yield:** 90 mg, 147 µmol, 84%.

^{1}H NMR (500 MHz, C_6D_6): δ = 1.37−1.49 (m, 6H, Ad-C*H*$_2$), 1.75−1.77 (m, 6H, Ad-C*H*$_2$), 1.78−1.82 (m, 3H, Ad-C*H*), 2.11 (s, 6H, C*H*$_3$-*para*), 2.40 (s, 6H, C*H*$_3$-*ortho*), 6.95 (s, 4H, Ar-*H*) ppm. **^{13}C{^{1}H} NMR** (126 MHz, C_6D_6): δ = 21.09 (s, *C*H$_3$-*para*), 28.56 (s, *C*H$_3$-*ortho*, Ad-C*H*), 36.74 (s, Ad-*C*H$_2$), 38.37 (s, Ad-C*H*$_2$), 62.02 (s, Ad-*C*), 129.75 (s, Ar*C*-meta), 137.44 (s, Ar*C*-para), 145.57 (s, Ar*C*-*ortho*), 157.12 (s, Ar*C*-*ipso*) 247.47 (s, C=O) ppm. **HR-MS (LIFDI, pos.):** calculated for $(^{12}C_{29}{}^{1}H_{37}{}^{209}Bi^{16}O)^+$: *m/z* = 610.26482, found: *m/z* = 610.26325. **UV-vis (DCM):** λ_{max} = 232 nm, 253 nm, 308 nm, 406 nm, onset at 482 nm. **Elemental analysis:** Anal. calc. (%) for $C_{29}H_{37}BiO$ (610.59 g · mol^{-1}): C 57.05, H 6.11; found: C 57.27, H 5.94. **IR (neat):** $\tilde{\nu}_{CO}$ = 1690 (s) cm^{-1}.

Synthesis of $C_{14}H_{10}$BiAd (**11**)

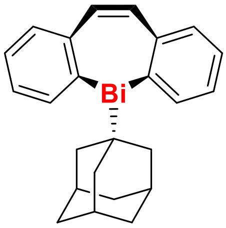

Method A: A solution of **9** (159 mg, 288 µmol) in toluene (14 mL) was stirred at rt for 24 h, during which a complete discoloration of the previously yellow solution occurred. The colorless solution was filtered, layered with *n*-pentane (10 mL) and stored at −30 °C. After 2 d **11** had precipitated as a colorless powder and was isolated by filtration, washed with *n*-pentane (3 x 2 mL) and dried *in vacuo*. **Yield:** 125 mg, 239 µmol, 83%.

Method B: In a J. Young NMR tube **9** (20 mg, 38 µmol) was dissolved in CD_2Cl_2. The yellow solution was irradiated with a blue LED (λ_{max} = 460 nm) for 25 min. A ^{1}H NMR spectrum of the now colorless solution revealed the formation **11** as the main product (traces of dibismuthane **1** were also detected). Spectroscopic yield: 81%.

^{1}H NMR (500 MHz, CD_2Cl_2): δ = 1.74−1.85 (m, 6H, Ad-C*H*$_2$), 1.98−2.03 (m, 3H, Ad-C*H*), 2.35 (d, 6H, $^3J_{HH}$ = 2.6 Hz, Ad-C*H*$_2$), 6.69 (s, 2H, *H*7/8), 7.26−7.30 (m, 4H, *H*3/4/11/12), 7.39−7.43 (m, 2H, *H*5/10), 7.86−7.90 (m, 2H, *H*2/13) ppm. **^{13}C{^{1}H} NMR** (126 MHz, CD_2Cl_2): δ = 33.70 (s, Ad-*C*) 37.36 (s, Ad-*C*), 45.98 (s, Ad-*C*), 57.87 (s, Ad-*C*), 127.73 (s, *C*3/12), 129.00 (s, *C*4/11), 133.17 (s, *C*5/10) 133.59 (s, *C*7/8), 138.22 (s, *C*2/13) 143.90 (s, *C*6/9), 151.32 (s, *C*1/14) ppm. **HR-MS (LIFDI, pos.):** calculated for $(^{12}C_{24}{}^{1}H_{25}{}^{209}Bi)^+$: *m/z* = 522.17601, found: *m/z* = 522.17607. **Elemental analysis:** Anal. calc. (%) for $C_{24}H_{25}Bi$ (522.44 g · mol^{-1}): C 55.18, H 4.82; found: C 55.54, H 4.88.

Synthesis of Mes_2BiAd (**12**)

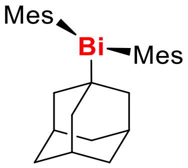

The synthesis was based on a literature procedure for the preparation of 1-adamantyl calcium halides.[6]

Elemental lithium (16 mg, 2.32 mmol) and biphenyl (359 mg, 2.32 mmol) were stirred in THF (10 mL) at rt for 3 h. The dark-green solution was added to a suspension of CaI_2 (341 mg, 1.16 mmol), and the reaction mixture was stirred for 1 h at rt. The dark-orange suspension was cooled to −78 °C and a solution of 1-bromoadamantane (250 mg, 1.16 mmol) in THF (7 mL) was added dropwise. After stirring for 20 min at −78 °C a suspension of dimesityl bismuth chloride (432 mg, 895 μmol) in a mixture of THF (10 mL) and toluene (4 mL) was added dropwise. The suspension was warmed to room temperature overnight, resulting in the formation of a black suspension. All volatiles were removed under reduced pressure, and the residue was extracted with *n*-pentane (10 mL) and *n*-hexane (4 mL). The solvent mixture was allowed to evaporate at −30 °C, which resulted in two batches of solid material after one and two days respectively. The solids were isolated by filtration and dried *in vacuo*. The first batch (151 mg, off-white powder) was identified as biphenyl via ^{1}H NMR spectroscopy. The second batch consisted of analytically pure, pale-orange crystals of **12**. **Yield:** 102 mg, 175 μmol, 20%.

^{1}H NMR (500 MHz, CD_2Cl_2): δ = 1.75−1.94 (m, 6H, Ad-C*H*$_2$), 2.03−2.08 (m, 3H, Ad-C*H*), 2.21 (s, 6H, C*H*$_3$-*para*), 2.23 (s, 12H, C*H*$_3$-*ortho*), 2.41−2.44 (m, 6H, Ad-C*H*$_2$), 6.94 (s, 4H, Ar-*H*) ppm. **^{13}C{^{1}H} NMR** (126 MHz, CD_2Cl_2): δ = 21.12 (s, *C*H$_3$-*para*), 28.87 (s, *C*H$_3$-*ortho*), 34.12 (s, Ad-*C*H), 37.26 (s, Ad-*C*H$_2$), 45.66 (s, Ad-*C*H$_2$), 60.48 (s, Ad-*C*) 129.25 (s, Ar*C*-*meta*), 137.00 (s, Ar*C*-*para*), 145.33 (s, Ar*C*-*ortho*), 161.65 (s, Ar*C*-*ipso*) ppm. **HR-MS (APCI, pos.):** calculated for $(^{12}C_{18}{}^{1}H_{22}{}^{209}Bi)^+$ (Mes_2Bi^+): *m/z* = 447.1520, found: *m/z* = 447.1507, calculated for $(^{12}C_{19}{}^{1}H_{26}{}^{209}Bi)^+$ ($MesAdBi^+$): *m/z* = 463.1833, found: *m/z* = 463.1821. The molecular ion peak was not detected in multiple experiments using the soft ionization methods APCI or LIFDI. **Elemental analysis:** Anal. calc. (%) for $C_{28}H_{37}Bi$ (582.58 g · mol^{-1}): C 57.73, H 6.40; found: C 58.07, H 6.05.

Single-Crystal X-ray Diffraction Analysis

Table S1: Selected crystal structure data of the structure determinations.

Data	**5**	**6**	**7**	**9**	**10***	**11**	**12**
Empirical formula	$C_{32}H_{46}BiKN_2O_6$	$C_{36}H_{58}BiKN_2O_6$	$C_{30}H_{46}BiKN_2O_6$	$C_{25}H_{25}BiO$	$C_{29}H_{37}BiO$	$C_{24}H_{25}Bi$	$C_{28}H_{37}Bi$
Formula weight (g·mol^{-1})	802.79	862.92	778.77	550.43	610.56	522.42	582.55
Temperature (K)	100.00						
Radiation	MoKα (λ = 0.71073)						
Crystal system	monoclinic	triclinic	monoclinic	orthorhombic	orthorhombic	orthorhombic	monoclinic
Space group	$P2_1/c$	$P\bar{1}$	$P2_1/c$	$P2_12_12_1$	*Pbca*	*Pnma*	$P2_1/n$
a (Å)	11.1854(5)	10.5271(17)	13.093(4)	10.4267(2)	16.8908(14)	20.819(3)	12.011(2)
b (Å)	21.2579(10)	11.6024(18)	12.885(6)	11.2011(2)	13.5199(10)	12.7085(19)	10.325(2)
c (Å)	13.9178(5)	15.944(3)	19.803(6)	16.5634(4)	21.1195(18)	7.0171(12)	19.162(5)
α (°)	90	96.100(7)	90	90	90	90	90
β (°)	91.6060(10)	91.001(11)	104.00(3)	90	90	90	91.474(10)
γ (°)	90	96.244(10)	90	90	90	90	90
Volume (Å^3)	3308.0(2)	1924.1(6)	3242(2)	1934.45(7)	4822.9(7)	1856.6(5)	2375.6(9)
Z	4	2	4	4	8	4	4
Calculated density (g·cm^{-3})	1.612	1.489	1.596	1.890	1.682	1.869	1.629
Absorbtion coefficient (mm^{-1})	5.499	4.733	5.609	9.126	7.330	9.499	7.433
F(000)	1608.0	876.0	1560.0	1064.0	2416.0	1008.0	1152.0
2Θ range for collection	4.822 to 52.152	3.894 to 49.994	3.806 to 49.996	4.616 to 54.994	4.314 to 51.998	3.912 to 54.996	3.956 to 51.99
Reflections collected	12342	64993	42759	21549	8571	27412	62716
Independent reflections	12342	6763	5706	4445	4743	2229	4671
Data/restraints/parameters	12342/0/380	6763/0/421	5706/0/361	4445/0/245	4743/0/286	2229/0/121	4671/0/268
Goodness-of-fit on F^2	1.073	1.114	1.183	1.031	1.093	1.115	1.074
Final R indexes (I>=2σ (I))	R_1 = 0.0377, wR_2 = 0.0678	R_1 = 0.0347, wR_2 = 0.0940	R_1 = 0.0327, wR_2 = 0.0568	R_1 = 0.0215, wR_2 = 0.0539	R_1 = 0.0364, wR_2 = 0.0900	R_1 = 0.0127, wR_2 = 0.0299	R_1 = 0.0224, wR_2 = 0.0416
Final R indexes (all data)	R_1 = 0.0463, wR_2 = 0.0705	R_1 = 0.0357, wR_2 = 0.0946	R_1 = 0.0386, wR_2 = 0.0580	R_1 = 0.0225, wR_2 = 0.0542	R_1 = 0.0476, wR_2 = 0.0954	R_1 = 0.0134, wR_2 = 0.0301	R_1 = 0.0285, wR_2 = 0.0428
Largest diff. peak/hole [e Å^{-3}]	1.42/-1.87	2.21/-0.83	1.00/-1.78	0.98/-1.17	0.87/-2.66	1.41/-0.72	1.30/-0.94
CCDC number	2447486	2447487	2447488	2447489	2447490	2447491	2488909

*: The structure of compound **10** was solved as a non-merohedral twin. Additional data processing was performed with HKLF5Tools.[7]

Table S2. Selected bond lengths [Å] and angles [°] of compounds **5**-**7**.

	**5**	**6**	**7**
Bi−C	2.259(5)/2.271(5)	2.307(5)/2.312(5)	2.273(4)/2.301(5)
C−Bi−C	92.36(18)	95.52(16)	97.88(16)

Table S3. Selected bonding parameters of compounds **9**, **10** and **11** (bond lengths in angstroms, angles in degrees).

	**9**	**10**	**11**	**12**
Bi−C_{aryl}	2.233(6)/2.245(6)	2.273(4)/2.273(5)	2.2557(19)	2.281(4)/2.297(3)
Bi−$C_{carbonyl/Ad}$	2.389(5)	2.405(5)	2.308(2)	2.347(3)
C=O	1.196(7)	1.211(7)	-	-
C_{aryl}−Bi−C_{aryl}	92.5(2)	101.80(17)	90.39(10)	97.88(13)
C_{aryl}−Bi−$C_{carbonyl/Ad}$	88.9(2)/89.3(2)	87.94(16)/104.96(17)	100.99(6)	92.98(12)/118.09(13)

Adamant-1-yldimesitylbismuthane **12** crystallizes in the monoclinic space group $P2_1/n$ with Z = 4 (Figure S1). The molecular structure shows the bismuth atom in a distorted trigonal-pyramidal coordination geometry (C−Bi−C, 92.98(12)−118.09(13)°), with Bi−C_{aryl} bond lengths (2.281(4)/2.297(3) Å) similar to those in the mesityl-substituted acylbismuthane **10** (2.273(4)/2.273(5) Å). The Bi−C_{Ad} bond length of 2.347(3) Å is slightly longer than that in the closely related bismepine species **11** (2.308(2) Å), most likely due to the higher steric demand of the mesityl substituents. The C10−Bi1−C19 angle (118.09(13)°) is exceptionally large, exceeding values reported for trivalent bismuthanes bearing three carbon-based substituents. A higher C−Bi−C angle has been reported for a diarylbismuth chloride bearing two bulky terphenyl substituents ((2,6-$Mes_2H_3C_6)_2$BiCl, 123.9(3)°).[8]

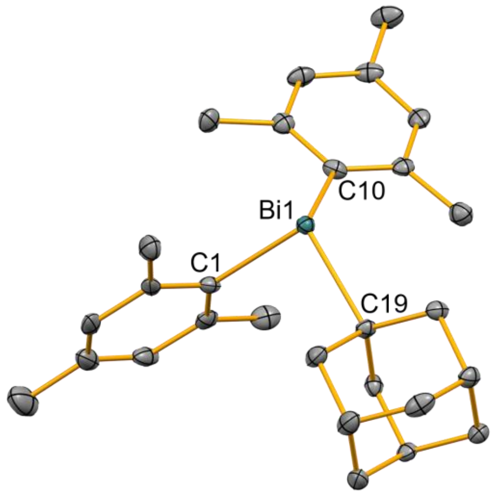

Figure S1. Molecular structure of compound **12** in the solid state (monoclinic space group $P2_1/n$, Z = 4). Displacement ellipsoids are shown at the 50% level. Hydrogen atoms are omitted for clarity. Selected bond lengths (Å) and angles (°): Bi1−C1, 2.281(4); Bi1−C10, 2.297(3); Bi1−C19, 2.347(3); C1−Bi1−C10, 97.88(13); C1−Bi1−C19, 92.98(12); C10−Bi1−C19; 118.09(13).

UV/Vis spectra

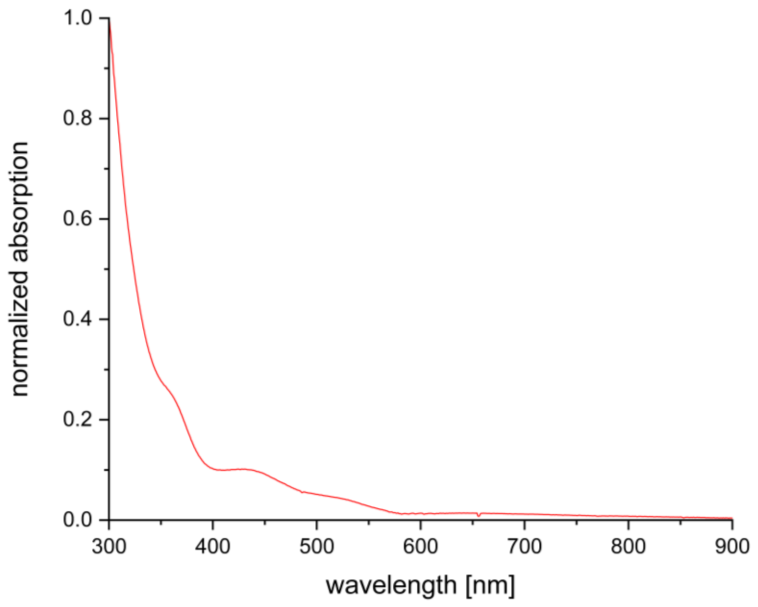

Figure S2: Experimental UV/Vis absorption spectrum of [K(crypt)][$C_{14}H_{10}$Bi] (**5**, 0.15 mM) in THF.

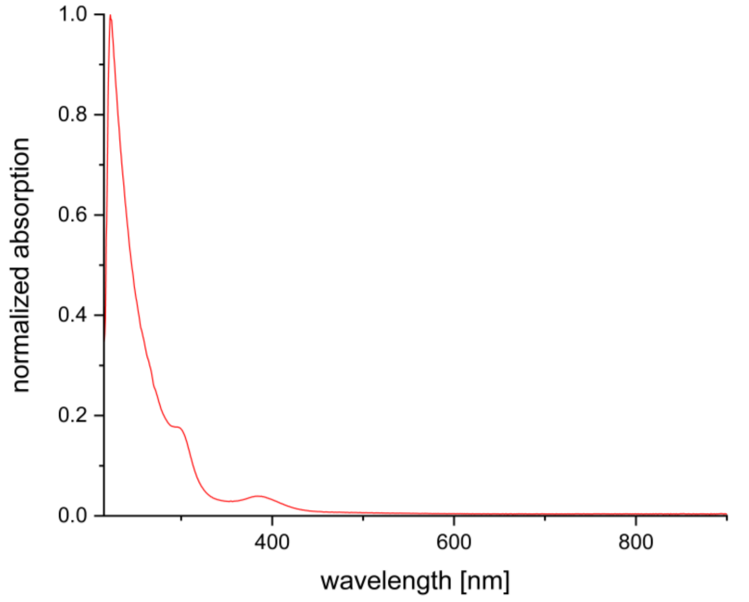

Figure S3: Experimental UV/Vis absorption spectrum of [K(crypt)][Mes_2Bi] (**6**, 0.06 mM) in THF.

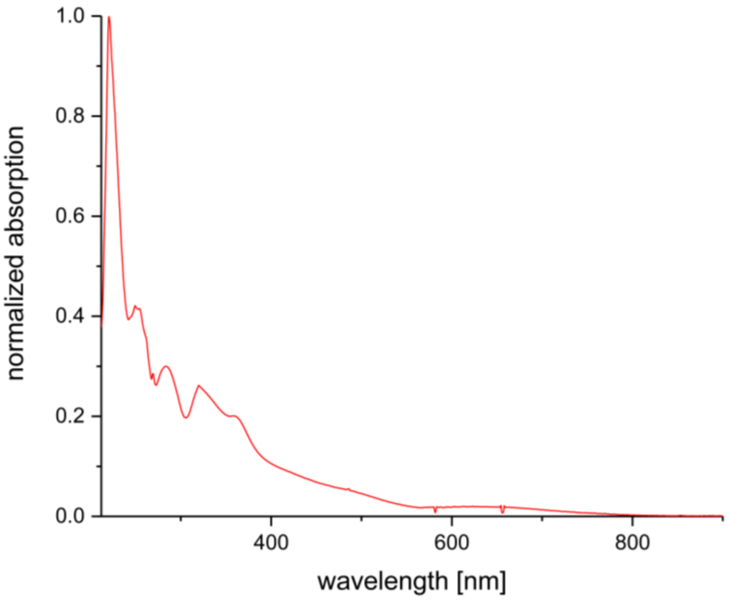

Figure S4: Experimental UV/Vis absorption spectrum of [K(crypt)][Ph_2Bi] (**7**, 0.08 mM) in THF.

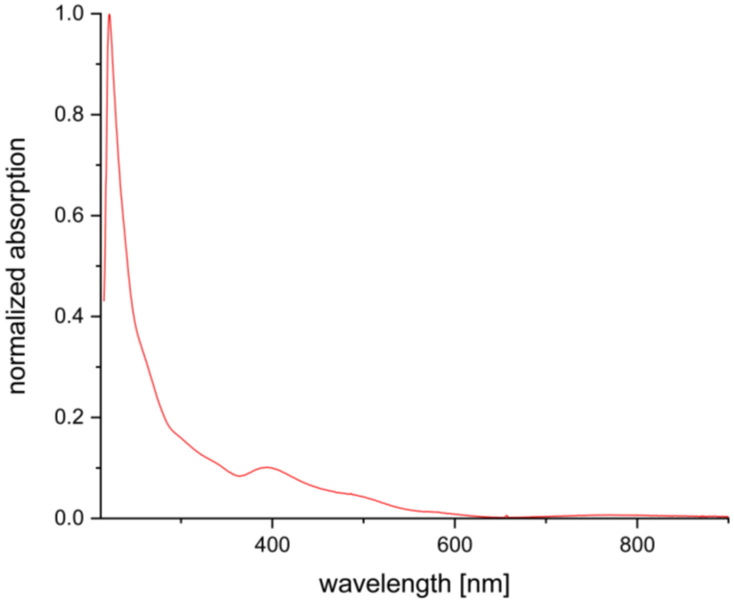

Figure S5: Experimental UV/Vis absorption spectrum of [K(crypt)][$Dipp_2$Bi] (**8**, 0.05 mM) in THF.

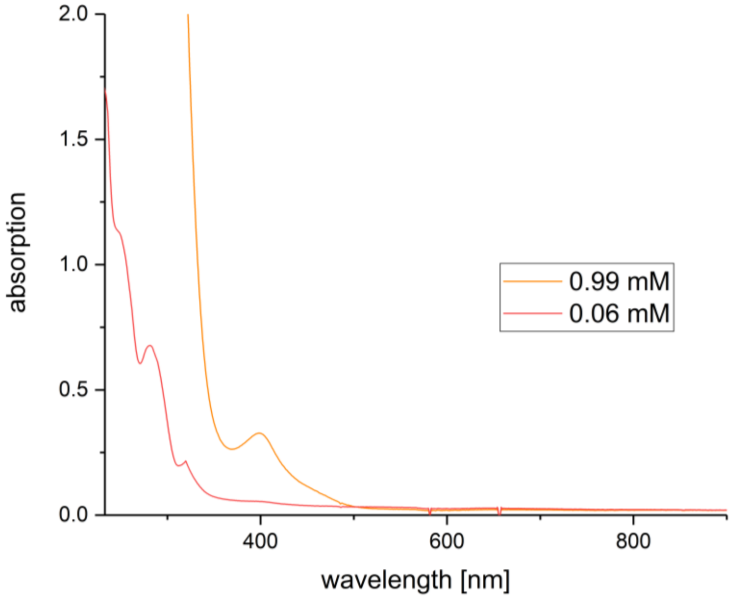

Figure S6: Experimental UV/Vis absorption spectra of $C_{14}H_{10}BiC(=O)Ad$ (**9**, orange: 0.99 mM, red: 0.06 mM) in dichloromethane.

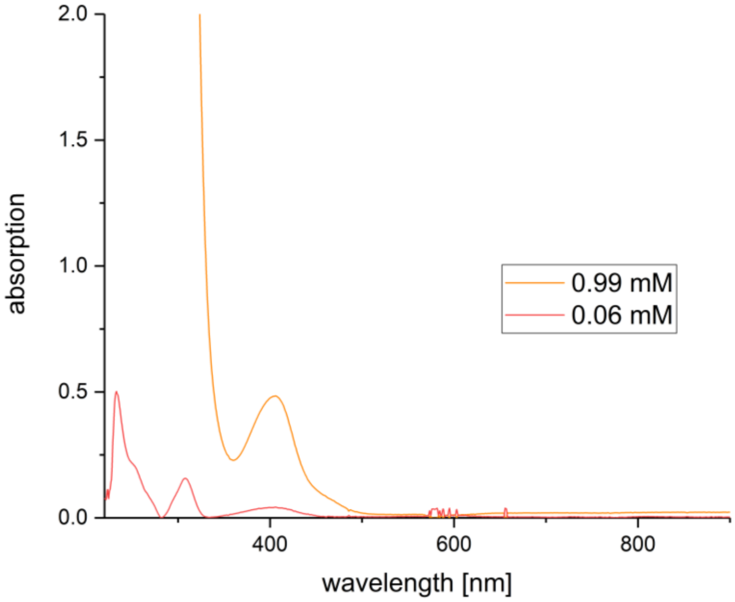

Figure S7: Experimental UV/Vis absorption spectra of $Mes_2BiC(=O)Ad$ (**10**, orange: 0.99 mM, red: 0.06 mM) in dichloromethane.

Thermochromism of diaryl bismuthides

Table S4: Overview of when thermochromism was observed upon cooling of compounds **5**-**8**.

	**5**	**6**	**7**	**8**
solid state	X	✓	X	✓
in solution (THF-d_8)	X	✓	X	X

As the thermochromism of some low-valent bismuth species has been noted in the literature,[4,9–14] compounds **5-8** were investigated with respect to this phenomenon. While compounds **5** and **7** did not show any color change upon cooling (either in solution or as solids), solid samples of **6** and **8** showed a color change from dark-red (at rt) to a pinkish red (after cooling with liquid N_2; Figures S8 and S9). An inverse color change from red (at rt) to a very dark orange (upon cooling with a dry ice/acetone bath) was observed for solutions of **6** in THF-d_8 (Figure S10). Under the same conditions, solutions of **8** did not show any color change. While some sources cite the formation of intermolecular Bi···Bi contacts as a possible explanation for the thermochromism of some bismuth compounds,[10–12,14] the absence of such contacts in the solid state structures of compounds **5-8** make this unlikely. The unequivocal identification of criteria causing the thermochromic behavior of compounds **6** and **8** or a general theory on when to expect such behavior remain to be established.

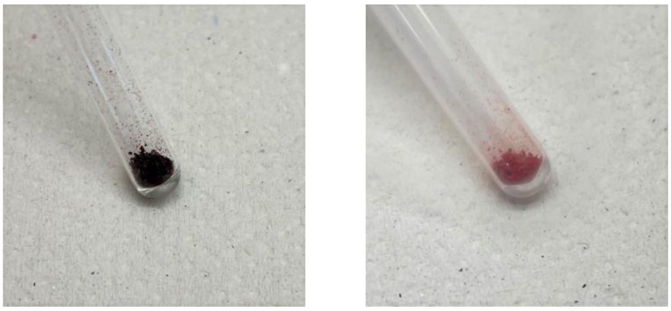

Figure S8: A solid sample of **6** at 20 °C (left) and after cooling to −196 °C (right).

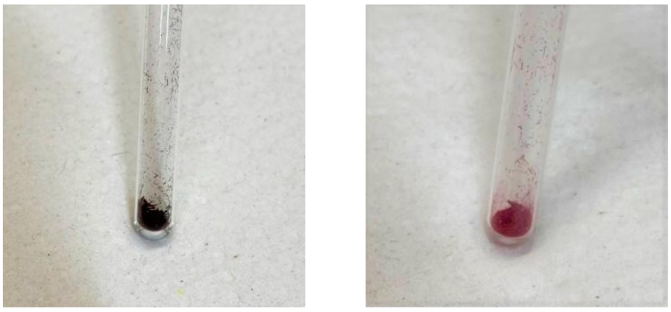

Figure S9: A solid sample of **8** at 20 °C (left) and after cooling to −196 °C (right).

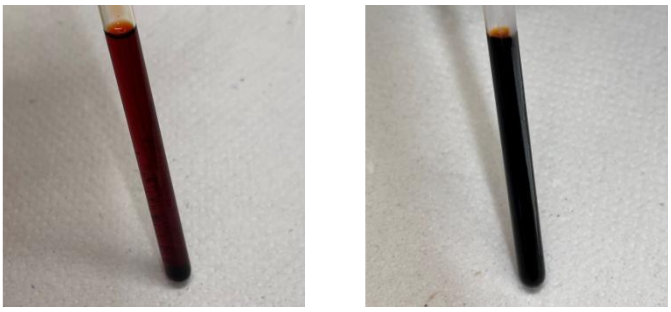

Figure S10: A solution of **6** in THF-d_8 (35 mM) at 20 °C (left) and after cooling to −78 °C (right).

Reactions with (^{13}C-labeled) carbon monoxide

Main group carbonyl and acyl species

Despite their fundamentally different electronic properties, the last 50 years have shown that (heavy) main-group elements can mimic the behavior of transition metals, including their ability to activate small molecules such as carbon monoxide.[15–20] Since Liebig's discovery that molten potassium reacts with carbon monoxide in 1834,[21] recent progress in main-group chemistry has shown that a variety of main-group species can activate CO.[22] Notable examples include alkali[23–27] and alkaline earth element species,[28–36] boron compounds,[37–46] frustrated Lewis pairs (FLPs),[47–54] heavier group 13 compounds,[55–59] carbenes[60–67] and their heavier analogues,[68–74] as well as phosphorous compounds.[75–78] Due to their lack of occupied orbitals of sufficient symmetry and energy for π-back-donation, the activation of carbon monoxide with heavier main-group elements (principal quantum number of n>4) has remained challenging. While tin and lead carbonyl species have been detected via IR spectroscopy in matrix isolation experiments, their isolation has remained elusive.[79–81] To the best of our knowledge, examples with an isolable product are limited to the reductive coupling of CO with a barium hydride complex bearing a bulky scorpionate ligand[82] and the insertion of CO into the Bi–N bond of a cationic bismuth amide reported by our group.[83,84] In most of these cases the activation of CO occurs irreversibly and only few reports describe the reversible activation of CO with main-group element compounds. The few literature-known examples can be divided into (i) the reversible coordination of CO to main-group element centers and (ii) the reversible insertion of CO into main-group element bonds. The reversible coordination of CO to borane species was first reported in 1937.[37] More recently the ability of two N,N'-diamidocarbenes,[60,61] a pentaarylborole,[39] a frustrated P/B Lewis pair,[50] a silylene[70] and a stannylene[74] to reversibly form main-group carbonyl complexes have been reported.

Reactions of the second type, i.e. the reversible insertion of CO into main-group element bonds are even more scarce and have been limited to three examples, with two of those involving group 13 compounds. In 2017 Braunschweig *et al.* reported the activation two CO molecules with a dihydrodiborene and the ability of the product to undergo reversible CO extrusion under formation of a B–B bond.[38] Two years later Crimmin *et al.* reported the reversible insertion of CO into the Al–C bond of a [2.2.1] aluminium metallobicycle leading to the formation of an acyl ligand attached to aluminium.[58] While the high instability of the product prohibited its structural characterization, its formation was confirmed via spectroscopic methods and the reversibility of the reaction demonstrated through isotopic exchange with ^{13}CO. In both examples the loss of CO is most likely induced thermally. Just recently recently the insertion of CO into the Si=Si bond of disilenes was reported, with only example being fully reversible (in the presence of a CO trapping agent) under thermal conditions.[85]

Despite its prevalence in transition metal carbonyls[86–91] and purely organic compounds (e.g. cyclic ketones),[92–98] reports on the light-induced decarbonylation of organometallic main-group compounds have remained rare.[70,75,99–101]

Acyl species of Si-Sn and P-Sb[102–115] have been explored in some detail, with a strong focus on Ge and P compounds, leading to intriguing reactivity patterns, perspectives towards applications in coating, printing inks, and health care,[112,113,116] as well as commercialized photoinitiators for radical polymerization.[117] In 1989 Kiyooka *et al.* reported the photochemical decarbonylation of α-arylacylgermanes using a high-pressure mercury lamp, which quantitatively leads to the formation of α-arylalkylgermanes.[101] To the best of our knowledge, additional examples of a photochemical decarbonylation of a main-group acyl species have not been reported to the literature. Harsh conditions using mercury lamp irradiation were reported in this case, whereas mild conditions exploiting visible light (LED or ambient light) are herein reported for this fundamental type of transformation.

Reactivity of **11** towards CO

In a J. Young NMR tube, **11** (13 mg, 25 µmol) was dissolved in CD_2Cl_2 (0.5 mL). The atmosphere above the solution was exchanged with CO (1.0 bar) *via* three freeze/pump/thaw cycles. The reaction was monitored via ^1H NMR spectroscopy and the sample stored at room temperature under ambient light between each measurement. Over the course of 4 d the formation of small amounts of acyl bismuthane **9** (up to approximately 6%), along with the formation of traces of other unidentified products, was observed and the amount of **9** in solution remained constant afterwards (monitored for up to 28 d). Similar results were obtained when other solvents (e.g. THF-d_8) were used and when the reaction was performed at elevated temperatures (up to 40 °C in CD_2Cl_2). Irradiation of a sample in CD_2Cl_2 that had reached equilibrium (i.e. 6% of compound **9**) with a blue LED (λ_{max} = 460 nm) for 1 h led to a decrease of the amount of **9** in solution (from 6% to 4%), concomitant with the formation of a small amount of dibismuthane **1** (approximately 3%) according to ^1H NMR spectroscopy. The formation of similar amounts of dibismuthane **1** as a side-product was also observed during the irradiation of a solution of **9** in CD_2Cl_2 (under an atmosphere of argon, not of CO), as described in the main part.

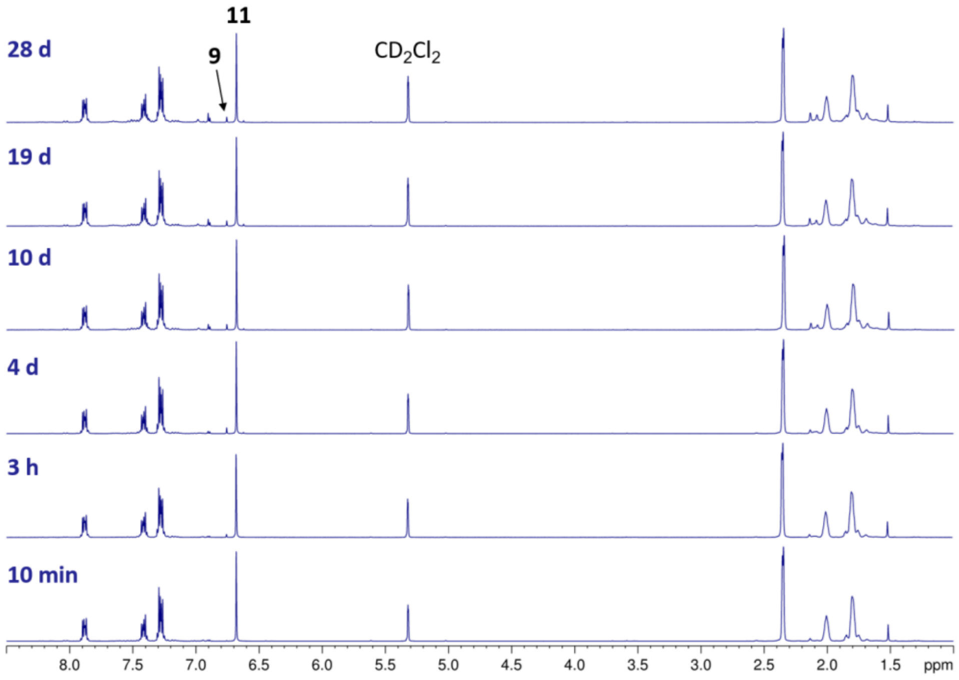

Figure S11: ^1H NMR spectra (500 MHz) of the reaction of **11** with CO in CD_2Cl_2 after 10 min, 3 h, 4 d, 10 d, 19 d and 28 d.

When a sample was prepared in the same way as described above (i.e. 10 mg **11** in CD_2Cl_2, 1.0 bar CO), but then stored under exclusion of light between ^{1}H NMR spectroscopic measurements (as far as possible), the slow formation of minor amounts of **9** was still observed. However, the formation of **9** was significantly slower (e.g.: 1% **9** after 24 h in the absence of light and 3% **9** after 3 h in the presence of light). In the absence of light (the reaction vessel was protected by a cover, which had to be removed for the insertion into the NMR spectrometer), the spectroscopic yield of **9** reached approximately 6% after 13 d and remained constant afterwards. These findings indicate the acceleration of the reaction under photochemical conditions (in this context, it should be noted that UV radiation from ambient light may contribute to the activation of **11** that is exposed to CO).

Reactivity of **12** towards CO

In a J. Young NMR tube, **12** (12 mg, 21 μmol) was dissolved in CD_2Cl_2 (0.5 mL). The atmosphere above the solution was exchanged with CO (1.0 bar) *via* three freeze/pump/thaw cycles. The reaction was monitored via ^1H NMR spectroscopy and the sample stored at room temperature under ambient light between each measurement. A ^1H NMR spectrum of the reaction mixture after 1 h showed the resonances of the starting material and multiple new resonance signals in the aromatic, as well as the aliphatic region, indicating an incomplete and unselective reaction (>10 products). While a longer reaction time (up to 48 h) led to a slight decrease of the intensity of the resonance signals assigned to **12**, the reaction remained incomplete, and the product distribution did not change significantly. The acyl bismuthane **10** was identified as one of the products by comparison of the ^1H and $^{13}C\{^1H\}$ NMR data obtained from the reaction with that of a pure sample.

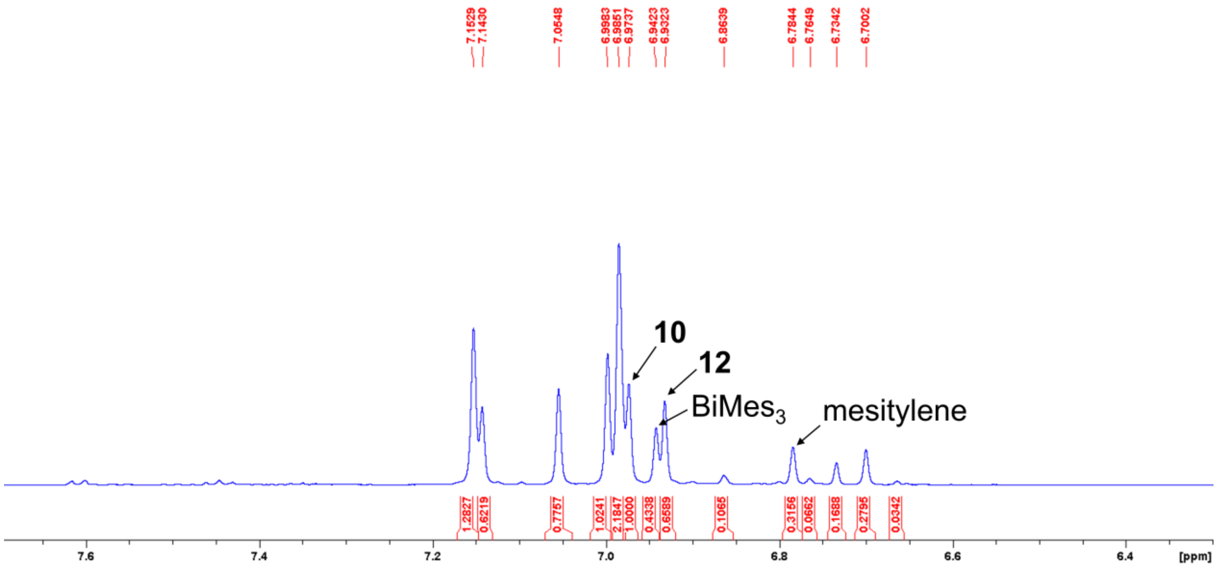

Figure S12: Aromatic region of the ^1H NMR spectrum (300 MHz) of the reaction of **12** with CO in CD_2Cl_2 after 48 h.

10 is formed in approximately 10% spectroscopic yield (value after 48 h, but only minor changes to this are observed at earlier stages of the reaction) and its formation was additionally confirmed by the detection of its molecular ion peak in a high-resolution mass spectrum of the reaction mixture (LIFDI, positive mode, Figure S13).

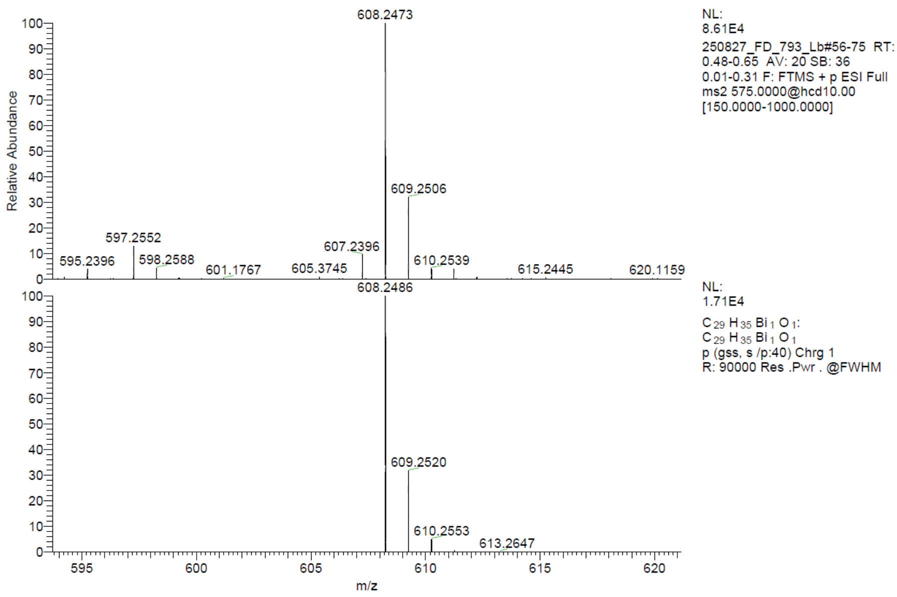

Figure S13: Experimental LIFDI-MS spectrum of the reaction mixture after 48 h (positive mode, top) and simulated isotope pattern of the cation [**10**]$^+$ (bottom).

Reactivity of 9 towards ^{13}CO

In a J. Young NMR tube, **9** (30 mg, 55 µmol) was dissolved in CD_2Cl_2 (0.5 mL). The atmosphere above the solution was exchanged with ^{13}CO (1.5 bar) *via* three freeze/pump/thaw cycles. The reaction was monitored via ^{1}H and ^{13}C{^{1}H} NMR spectroscopy and the sample stored at room temperature under ambient light between each measurement. The amount of compound **9** remaining was approximated *via* integration of the respective ^{1}H NMR spectroscopic signal assigned to the olefin units (H7/8) of compounds **9** and **11**. To gain insights into the incorporation of ^{13}CO into **9**, the relative intensity of the ^{13}C{^{1}H} NMR resonance signal assigned to the carbonyl C atom of **9** was determined using the solvent signal as a reference (integral set to a value of 1.00, Table S5).

Table S5: Data obtained from ^{1}H and ^{13}C{^{1}H} NMR spectra of the reaction of **9** with ^{13}CO in CD_2Cl_2.

	**30 min**	**25 h**	**48 h**	**120 h**	**288 h**	**388 h**
amount of **9** remaining [%]	95	35	23	19	17	16
^{13}C NMR integral carbonyl group of **9**	0.08	0.46	0.43	0.38	0.34	0.31
^{13}C NMR integral carbonyl group of **9** relative to amount of **9** (normalized)	0	0.64	0.93	1	1	0.97

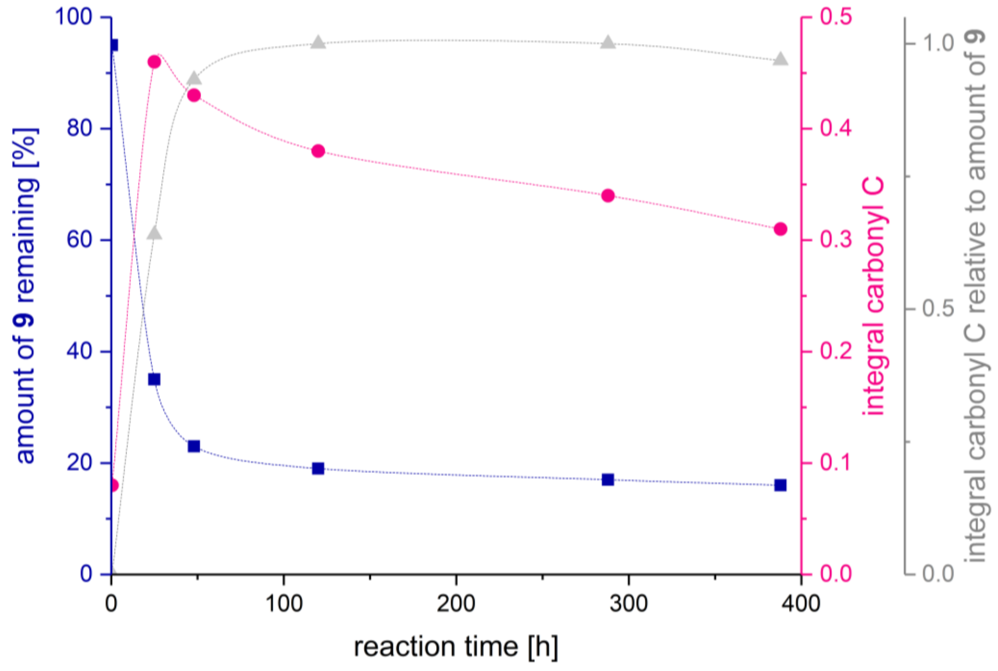

Figure S14: Plot of the amount of **9** remaining (blue squares), the intensity of the ^{13}C NMR spectroscopic resonance of the carbonyl group of **9** (pink dots) and the intensity of the ^{13}C NMR spectroscopic resonance of the carbonyl group of **9** relative to the remaining amount of **9** (normalized, grey triangles) against the reaction time with dotted trendlines shown as guide to the eye.

Despite the atmosphere of ^{13}CO (1.5 bar), CO extrusion under formation of **11** is still observed under these conditions, albeit at a much slower rate and without complete consumption of the acyl bismuthane **9** (under Ar atmosphere, complete conversion is achieved after approximately 40 h). After 25 h, a sixfold increase of the intensity of the ^{13}C NMR spectroscopic resonance assigned to the carbonyl group of **9** (δ = 253.81 ppm) was observed. In the same spectra, the remaining resonances assigned to **9** decrease in intensity due to the decreasing amount of **9** in solution (to give **11**). The absolute and relative increase of the intensity of the signal due to the carbonyl group in **9** indicates that ^{13}CO is incorporated into compound **9** and is further proof for the reversibility of the CO extrusion. When the same reaction was performed and the sample stored under exclusion of light between measurements (as far as possible), no signs for CO extrusion or the incorporation of ^{13}CO into compound **9** were observed, giving further evidence of the light-driven nature of the reaction.

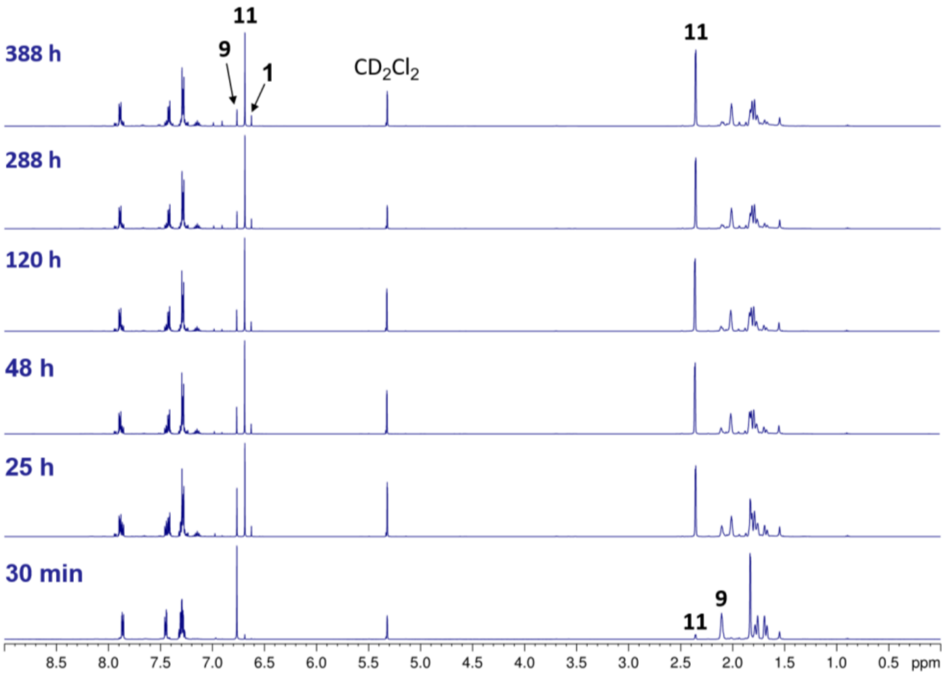

Figure S15: ^{1}H NMR spectra (500 MHz) of the reaction of **9** with ^{13}CO in CD_2Cl_2 after 30 min, 25 h, 48 h, 120 h, 288 h and 388 h.

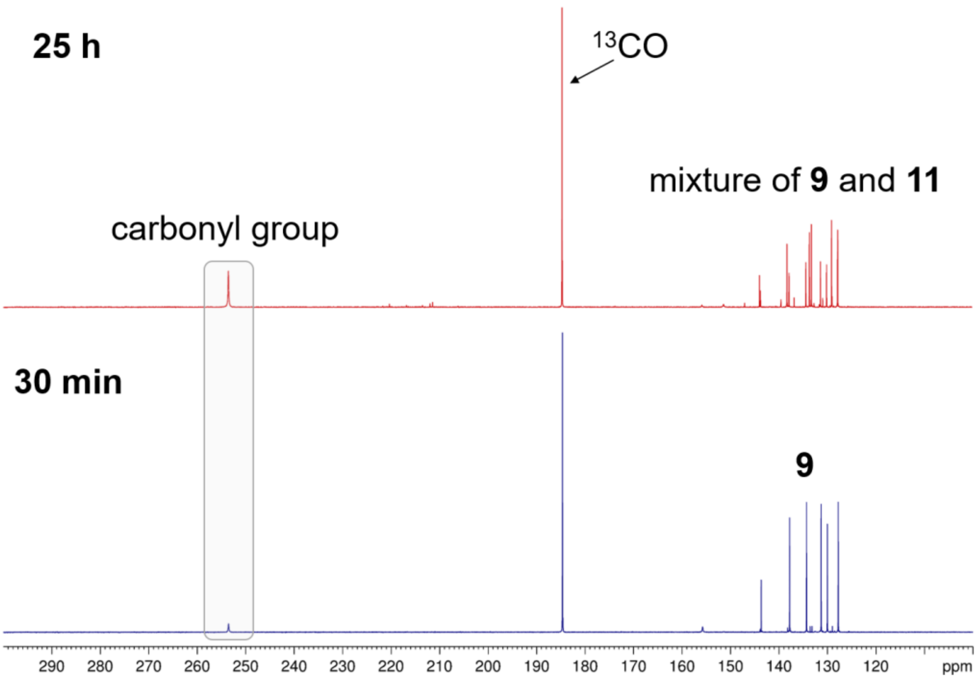

Figure S16: ^{13}C{^{1}H} NMR spectra (126 MHz) of the reaction of **9** with ^{13}CO in CD_2Cl_2 after 30 min and 25 h. The resonance assigned to the carbonyl group of **9** is highlighted by a grey box.

Reactivity of **10** towards ^{13}CO

In a J. Young NMR tube, **10** (15 mg, 25 µmol) was dissolved in CD_2Cl_2 (0.5 mL). The atmosphere above the solution was exchanged with ^{13}CO (1.5 bar) *via* three freeze/pump/thaw cycles. The reaction was monitored via ^{1}H and $^{13}C\{^{1}H\}$ NMR spectroscopy and the sample stored at room temperature under ambient light between each measurement. To gain insights into the incorporation of ^{13}CO into **10**, the relative intensity of the $^{13}C\{^{1}H\}$ NMR resonance signal assigned to the carbonyl C atom of **10** was determined using the solvent signal as a reference (integral set to a value of 1.00, Table S6).

Table S6: Data obtained from ^{1}H and $^{13}C\{^{1}H\}$ NMR spectra of the reaction of **10** with ^{13}CO in CD_2Cl_2.

	**before**	**30 min**	**24 h**	**7 d**	**9 d**	**10 d**
^{13}C NMR integral carbonyl group of **10**	0.015	0.050	0.318	0.463	0.466	0.519

After 24 h, a 21-fold increase of the intensity of the ^{13}C NMR spectroscopic resonance assigned to the carbonyl group of **10** (δ = 247.47 ppm) was observed, indicating that ^{13}CO is incorporated into compound **10** and proving the reversibility of the CO extrusion. Over the course of 10 d the intensity of the resonance signal continued to increase up to a factor of about 35 (when compared to a $^{13}C\{^{1}H\}$ NMR spectrum of the same sample before the addition of ^{13}CO), after which no further increase was observed. During the reaction, the slow formation of small amounts of mesitylene, $BiMes_3$ and other unidentified products was observed spectroscopically with about 73% of **10** being detected after 10 d. This indicates a slow and unselective degradation of **10** in solution.

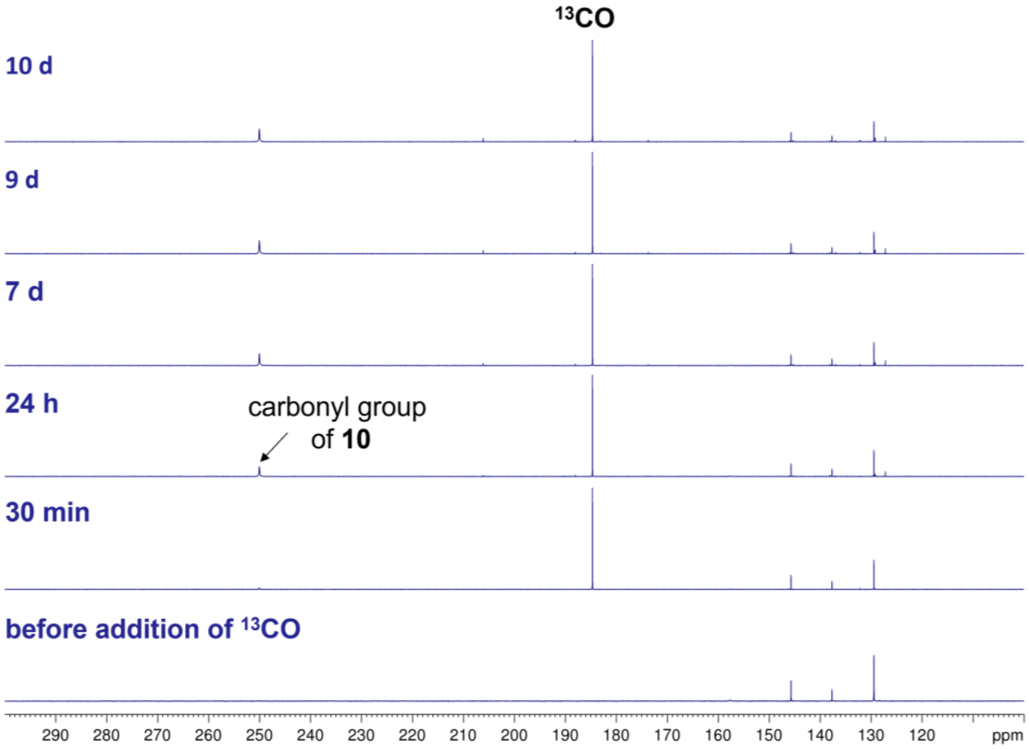

Figure S17: $^{13}C\{^{1}H\}$ NMR spectra (126 MHz) of the reaction of **10** with ^{13}CO in CD_2Cl_2 before the addition of ^{13}CO, after 30 min, 24h, 7d, 9 d and 10 d.

IR spectra

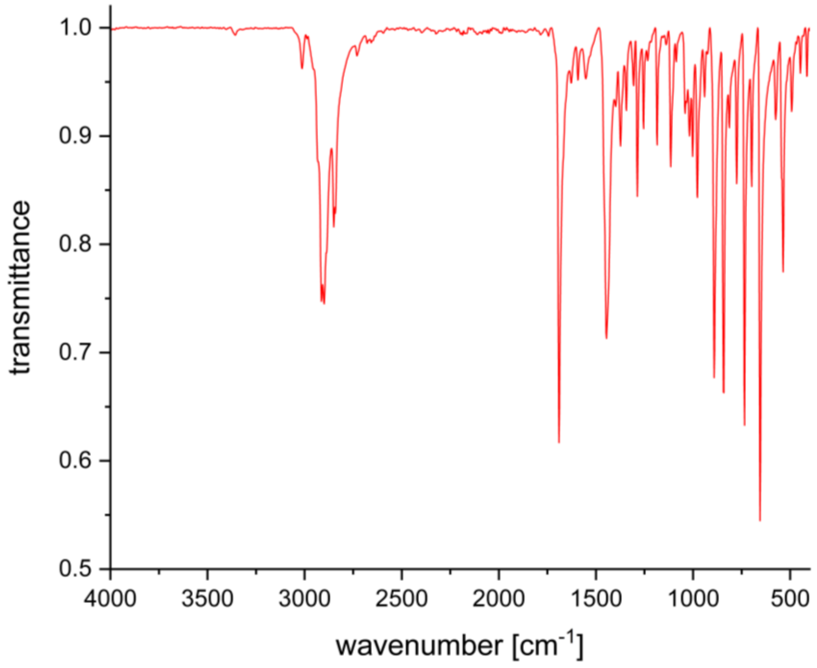

Figure S18: IR spectrum of $Mes_2BiC(=O)Ad$ (**10**, neat).

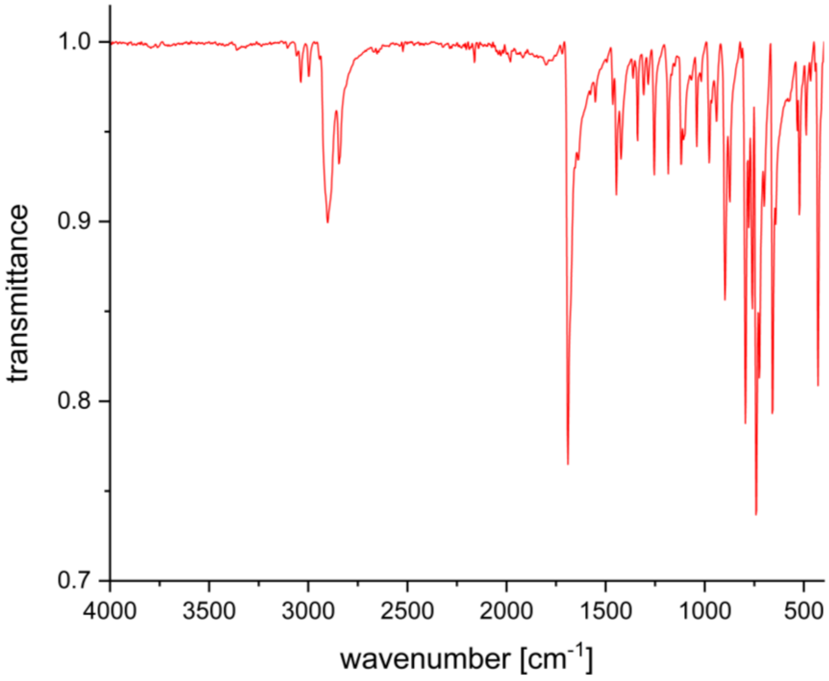

Figure S19: IR spectrum of $C_{14}H_{10}BiC(=O)Ad$ (**9**, neat).

An IR spectrum of **11** was measured to confirm the absence of a CO stretching vibration. As expected, the IR spectrum shows great similarity to that of compound **9** with the difference that no absorption was found in the expected range for CO stretching vibrations.

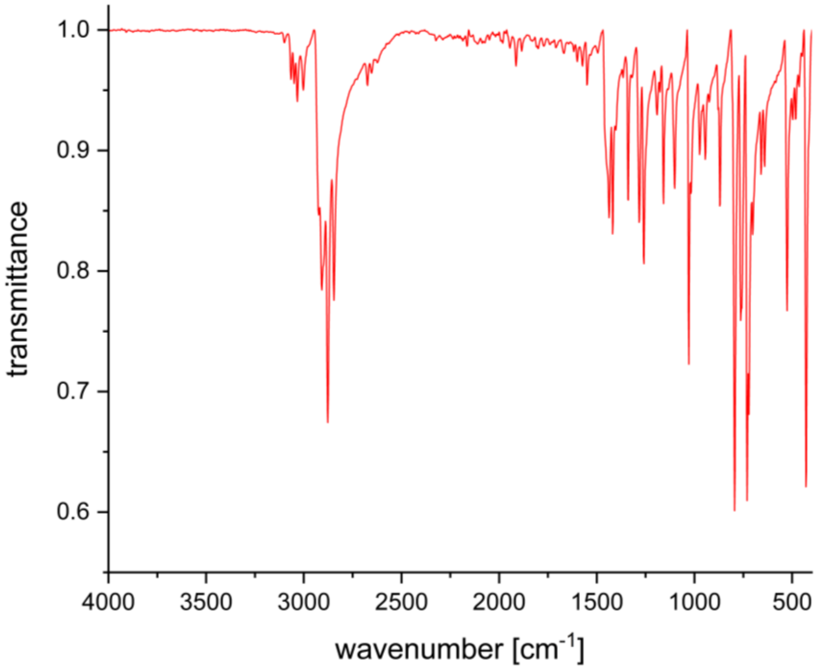

Figure S20: IR spectrum of $C_{14}H_{10}BiAd$ (**11**, neat).

NMR spectra

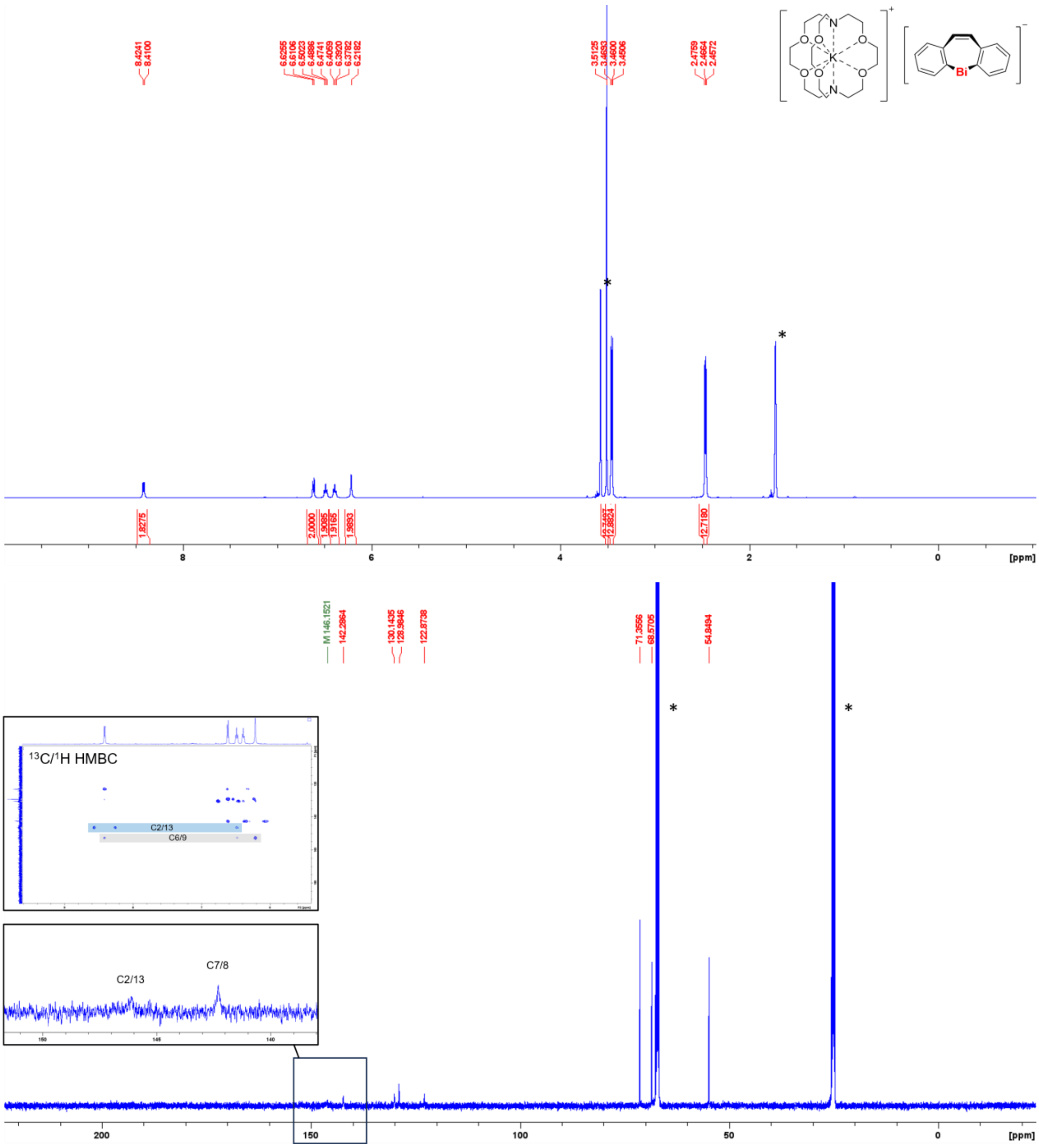

Figure S21: ^{1}H (top, 500 MHz) and ^{13}C{^{1}H} (bottom, 126 MHz) NMR spectra of **5** in THF-d_8 (*: solvent). The presence of the weak resonance signal at 146.15 ppm in the ^{13}C{^{1}H} NMR spectrum was additionally confirmed via ^{13}C/^{1}H HMBC experiments (upper black box, correlation peaks involving C2/13 are highlighted in light blue).

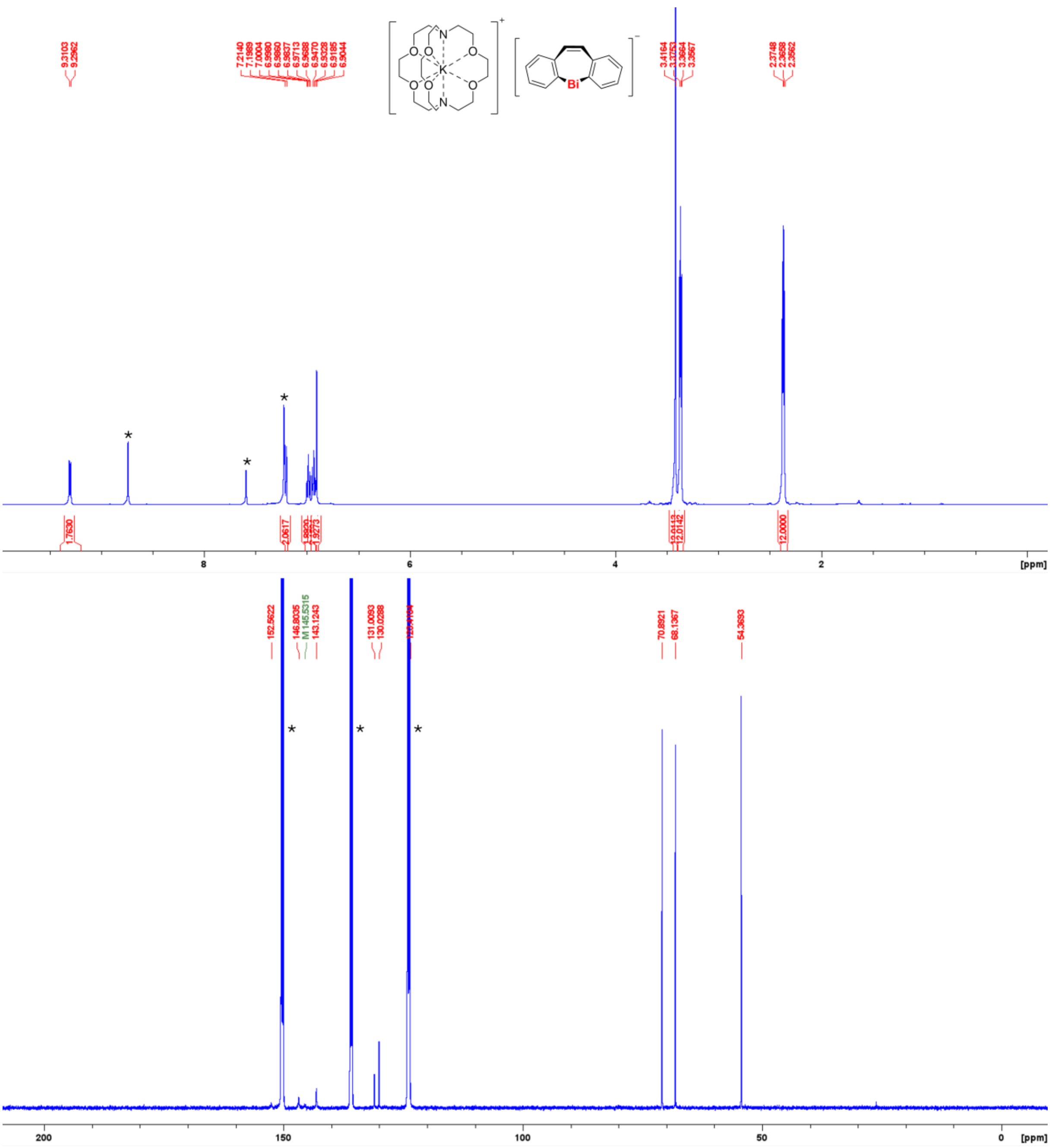

Figure S22: ^{1}H (top, 500 MHz) and ^{13}C{^{1}H} (bottom, 126 MHz) NMR spectra of **5** in pyridine-d_5 (*: solvent).

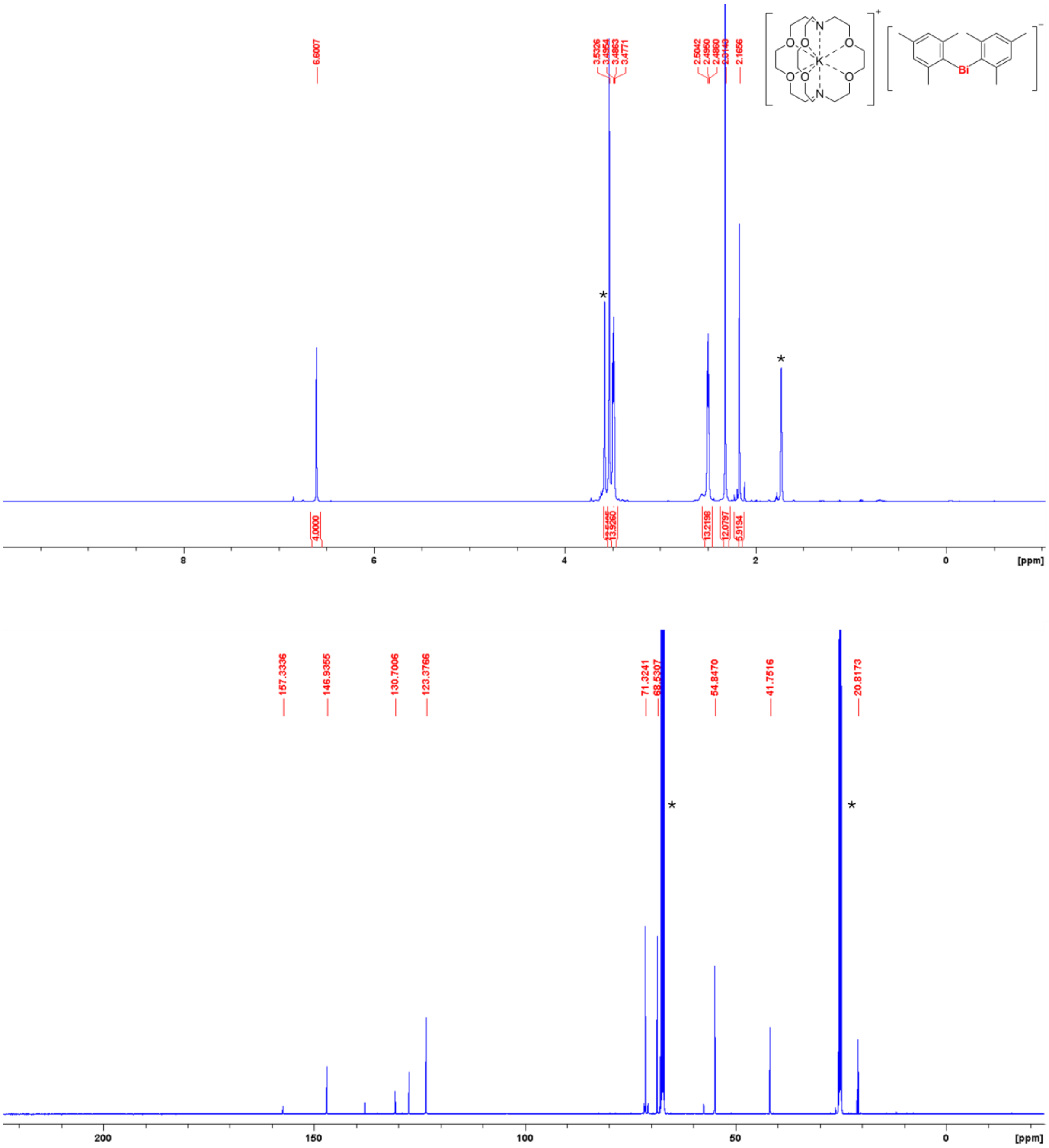

Figure S23: ^{1}H (top, 500 MHz) and ^{13}C{^{1}H} (bottom, 126 MHz) NMR spectra of **6** in THF-d_8 (*: solvent).

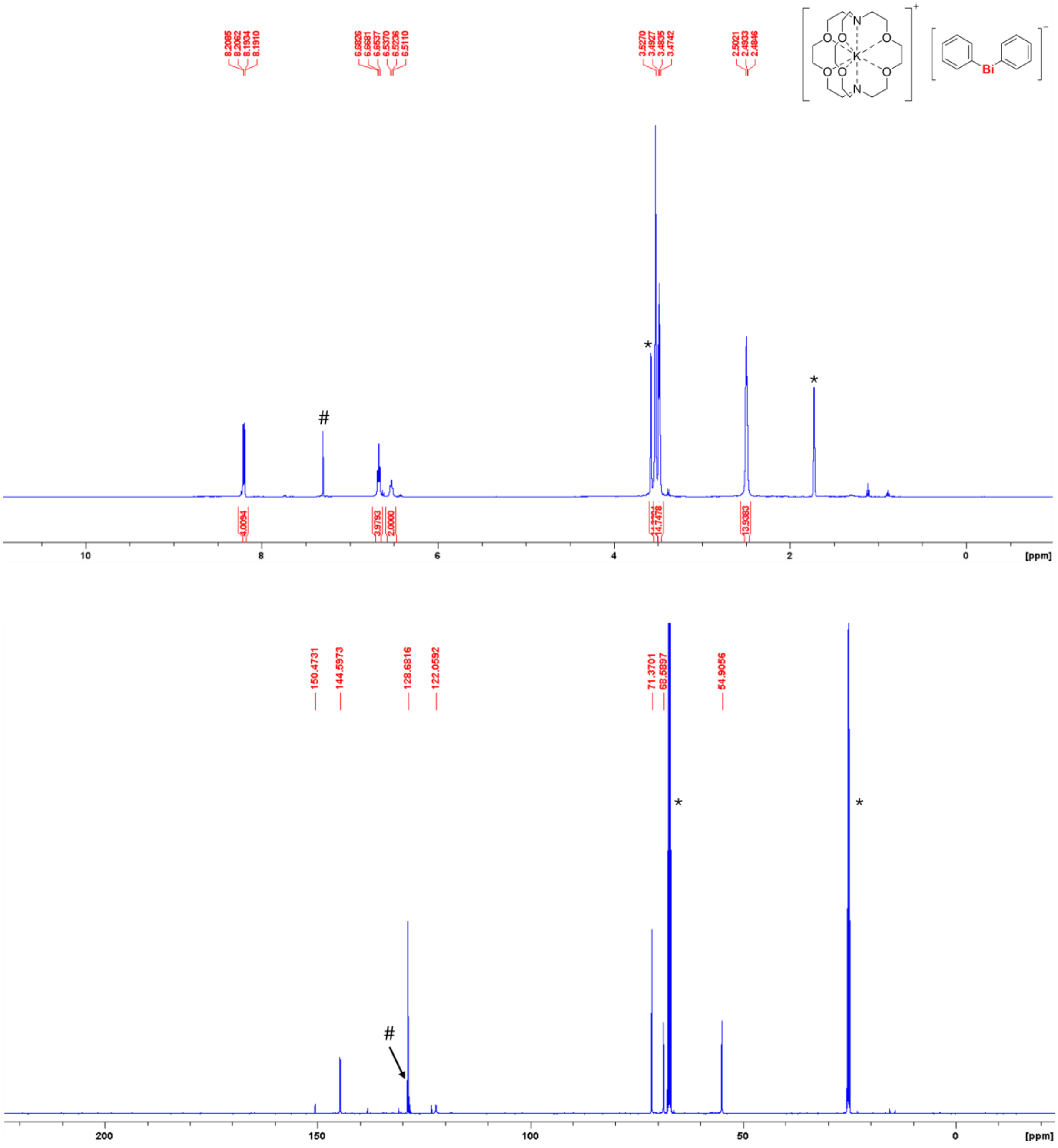

Figure S24: ^{1}H (top, 500 MHz) and ^{13}C{^{1}H} (bottom, 126 MHz) NMR spectra of **7** in THF-*d*$_8$ (*: solvent). The sample contained traces of benzene (#) due to the slow decomposition of **7** in solution.

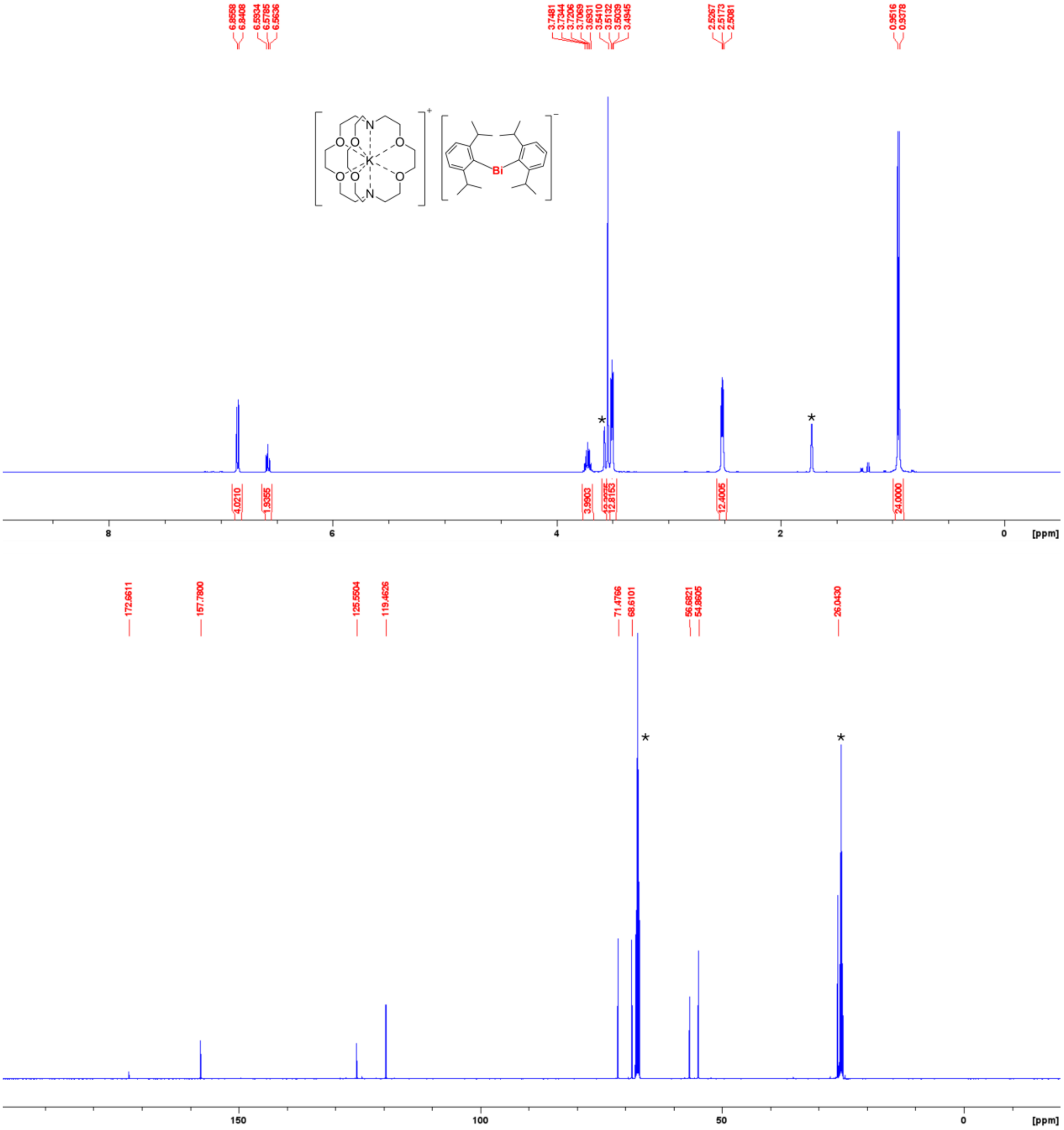

Figure S25: ^1H (top, 500 MHz) and $^{13}C\{^1H\}$ (bottom, 126 MHz) NMR spectra of **8** in THF-d_8 (*: solvent).

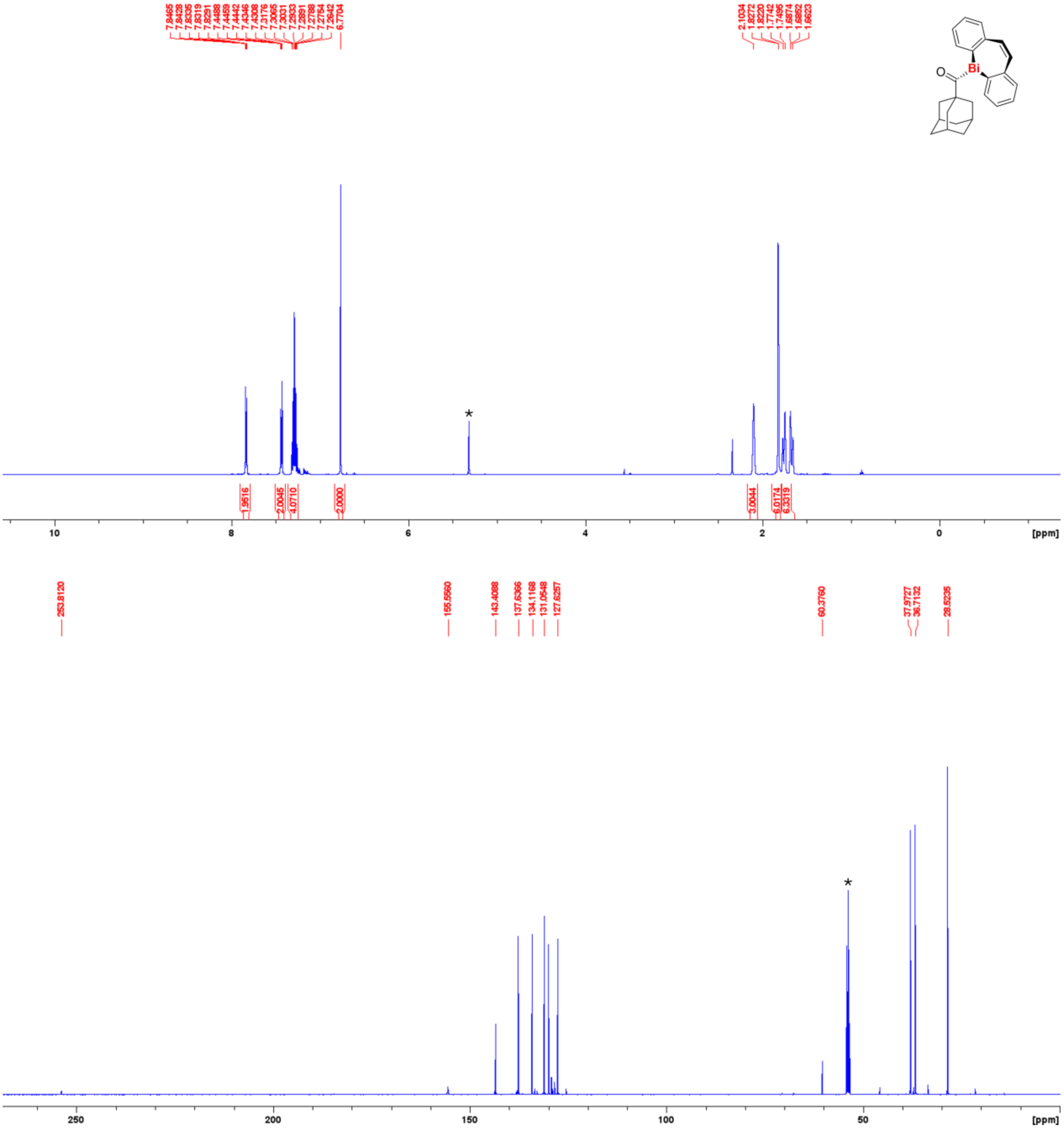

Figure S26: ^1H (top, 500 MHz) and $^{13}C\{^1H\}$ (bottom, 126 MHz) NMR spectra of **9** in CD_2Cl_2 (*: solvent).

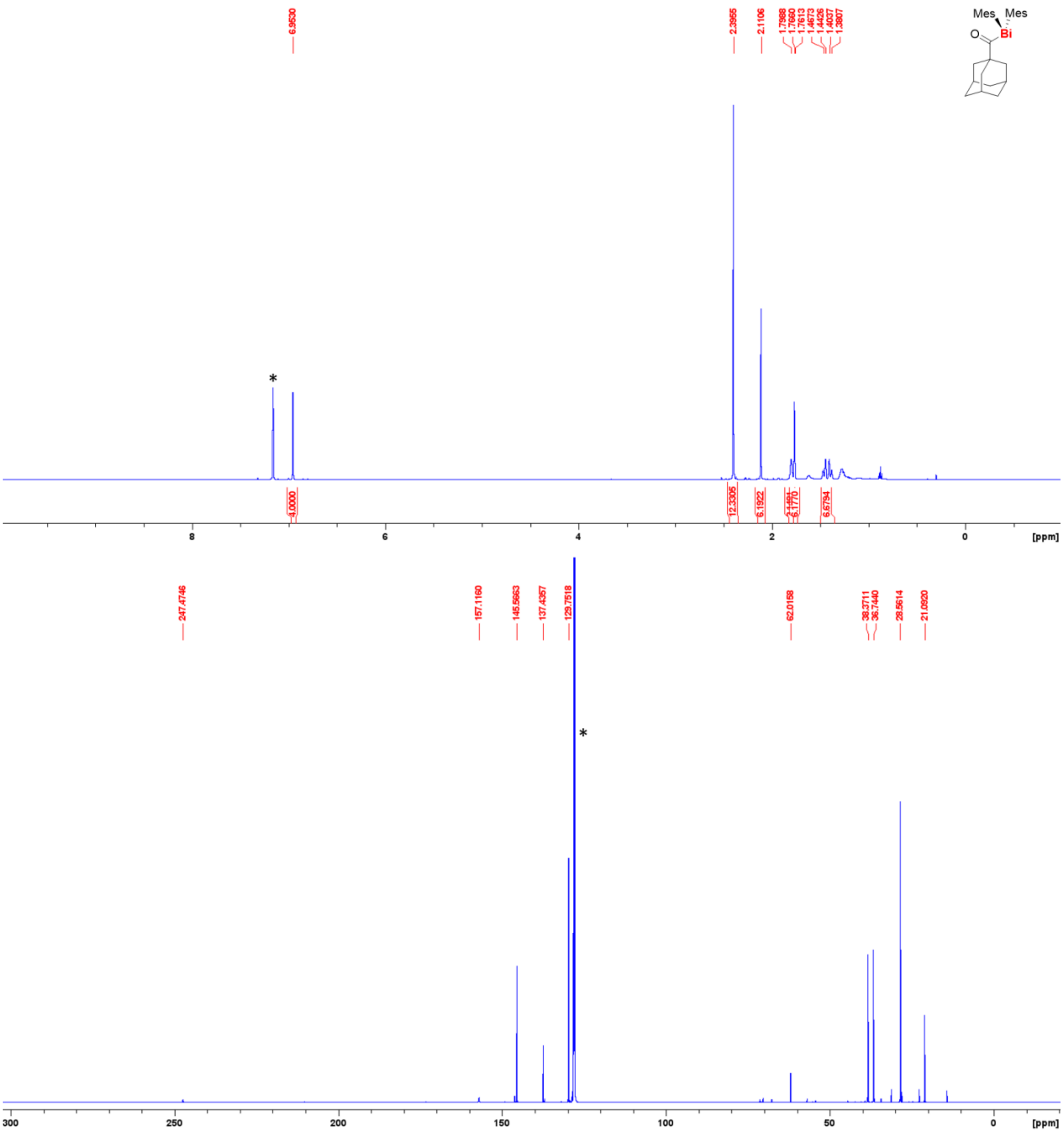

Figure S27: ^{1}H (top, 500 MHz) and $^{13}C\{^{1}H\}$ (bottom, 126 MHz) NMR spectra of **10** in C_6D_6 (*: solvent).

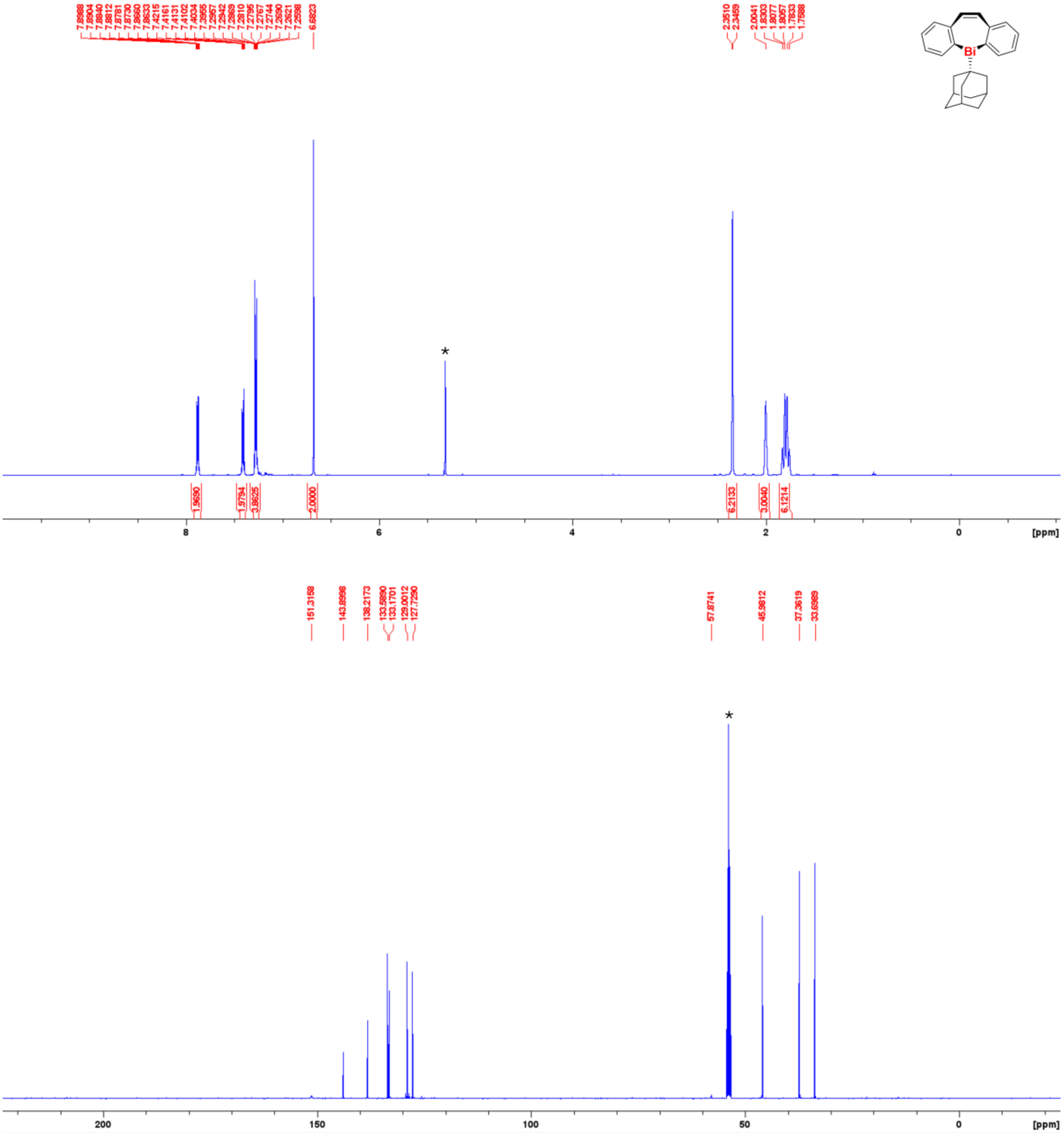

Figure S28: ^{1}H (top, 500 MHz) and $^{13}C\{^{1}H\}$ (bottom, 126 MHz) NMR spectra of **11** in CD_2Cl_2 (*: solvent).

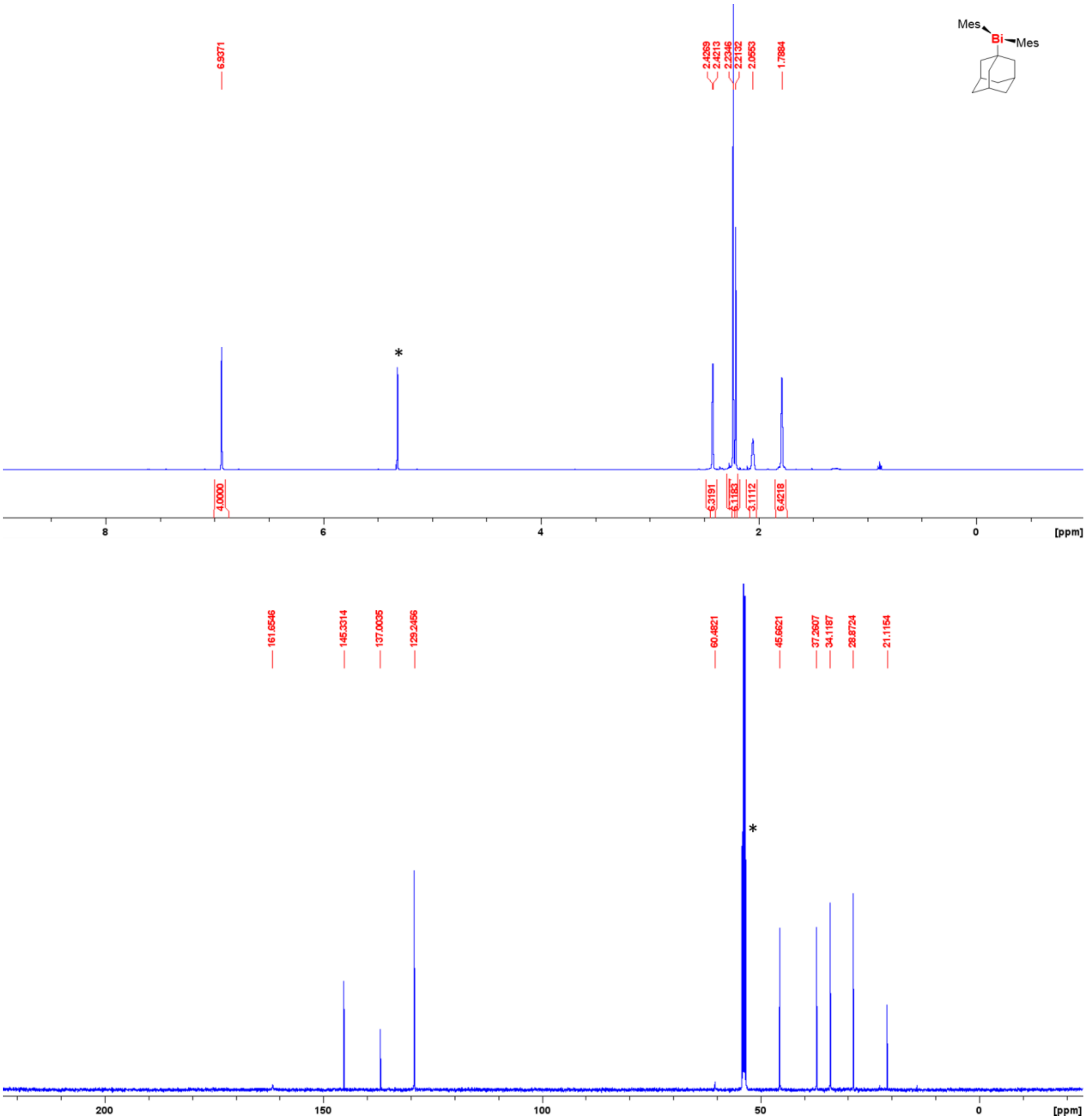

Figure S29: ^{1}H (top, 500 MHz) and $^{13}C\{^{1}H\}$ (bottom, 126 MHz) NMR spectra of **12** in CD_2Cl_2 (*: solvent).

Mass spectra

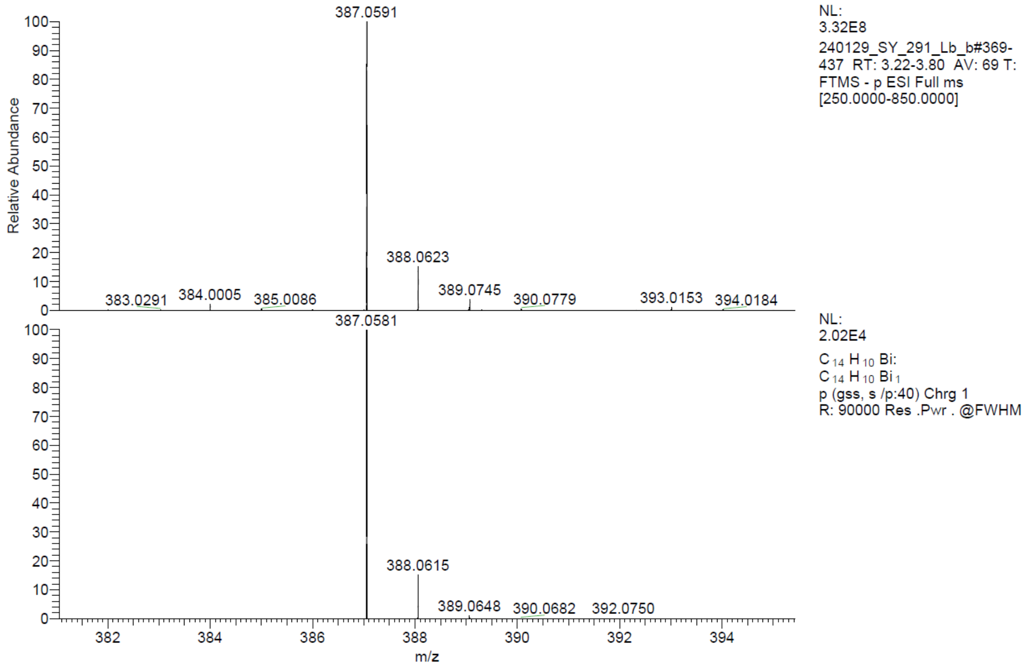

Figure S30: Experimental ESI-MS spectrum of compound **5** (negative mode, top) and simulated isotope pattern of the anion $C_{14}H_{10}Bi^{-}$ (bottom).

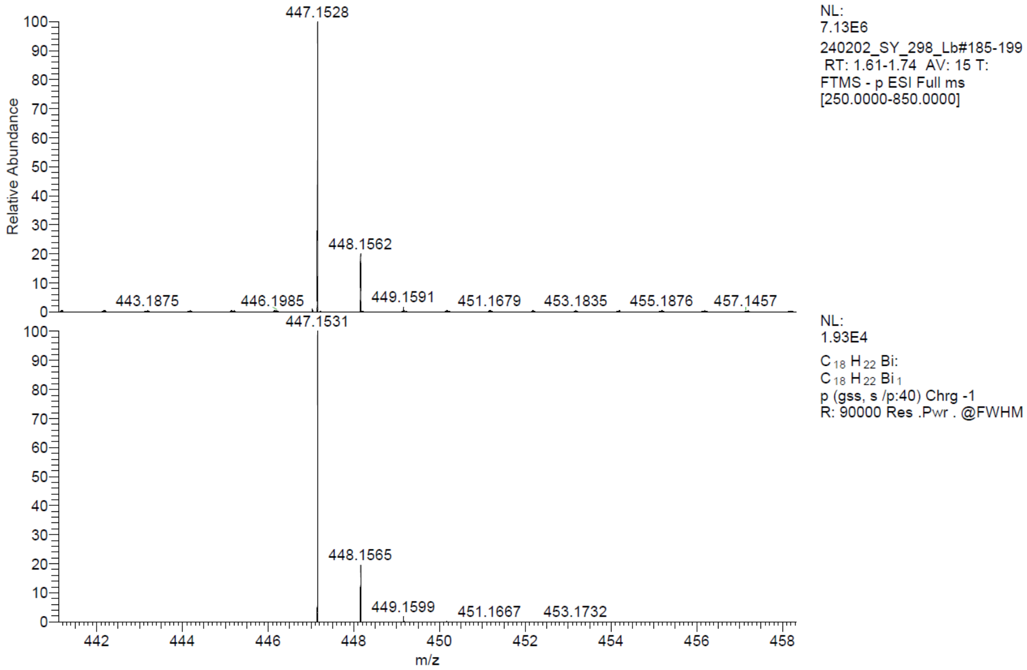

Figure S31: Experimental ESI-MS spectrum of compound **6** (negative mode, top) and simulated isotope pattern of the anion Mes_2Bi^{-} (bottom).

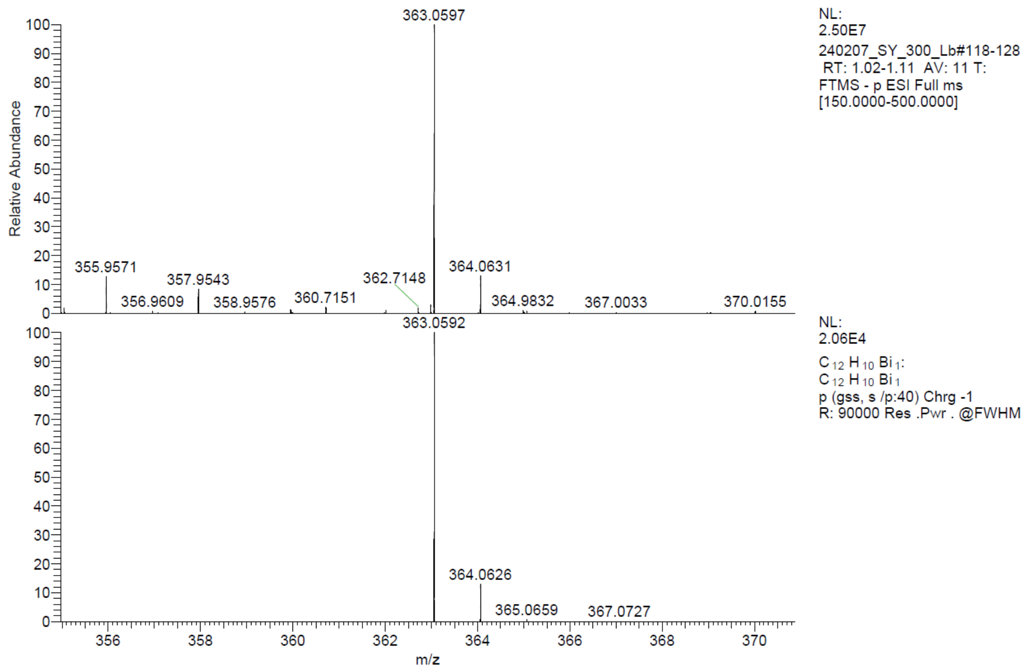

Figure S32: Experimental ESI-MS spectrum of compound **7** (negative mode, top) and simulated isotope pattern of the anion Ph_2Bi^- (bottom).

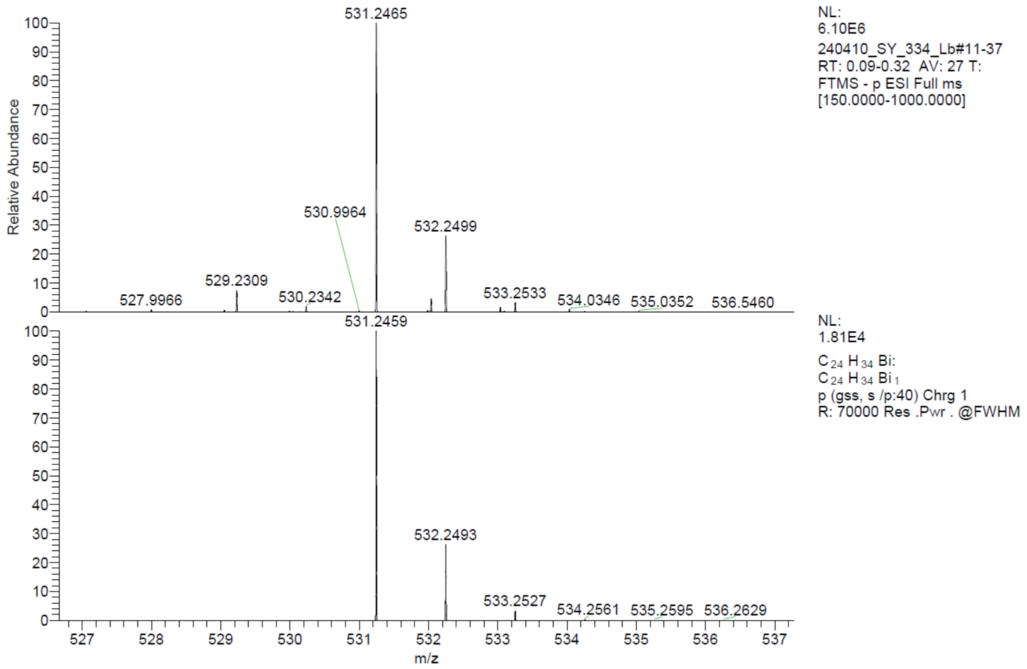

Figure S33: Experimental ESI-MS spectrum of compound **8** (negative mode, top) and simulated isotope pattern of the anion $Dipp_2Bi^-$ (bottom).

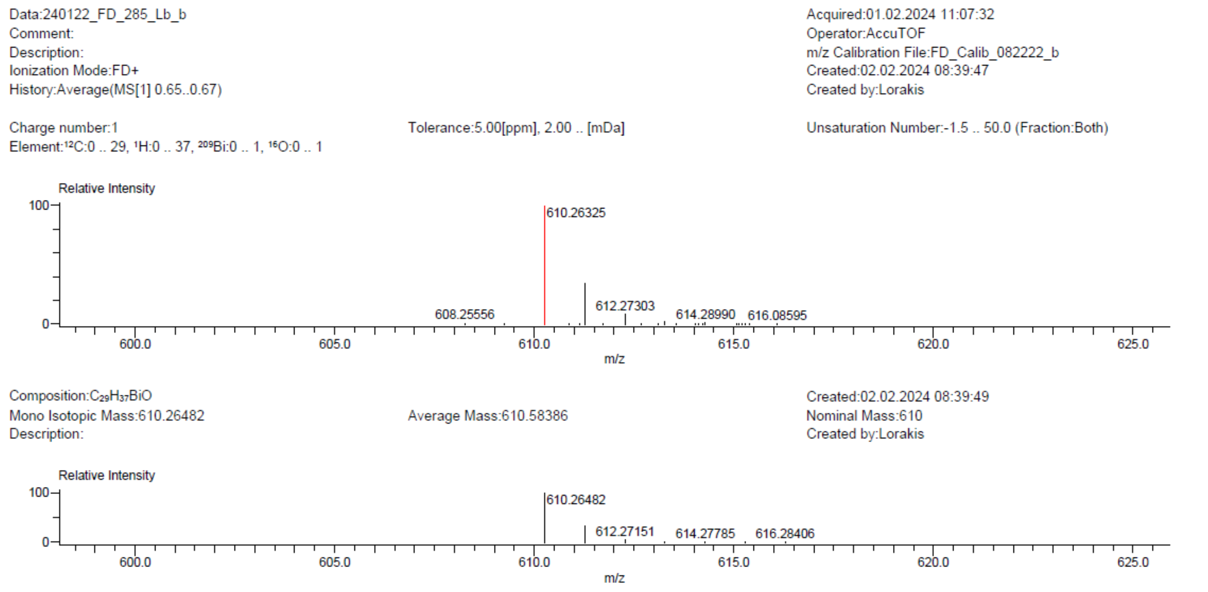

Mass	Intensity	Relative Intensity	Calc. Mass	Mass Difference [mDa]	Mass Difference [ppm]	Possible Formula	Unsaturation Number
610.26325	225959.32	100.00	610.26482	-1.57	-2.58	$^{12}C_{29}{}^{1}H_{37}{}^{209}Bi_{1}{}^{16}O_{1}$	12.0

Figure S34: Experimental LIFDI-MS spectrum of compound **10** (positive mode, top) and simulated isotope pattern of the cation [**10**]$^+$ (bottom).

Despite the soft nature of FD ionization, LIFDI-MS spectra of compound **9** only showed the molecular ion ([**9**]$^+$) as a minor species. Instead mainly peaks of ions resulting from the decomposition/fragmentation of **9** were detected (Figure S35). This was rationalized to be a result of the weak nature of the Bi−C$_{carbonyl}$ bond in compound **9**.

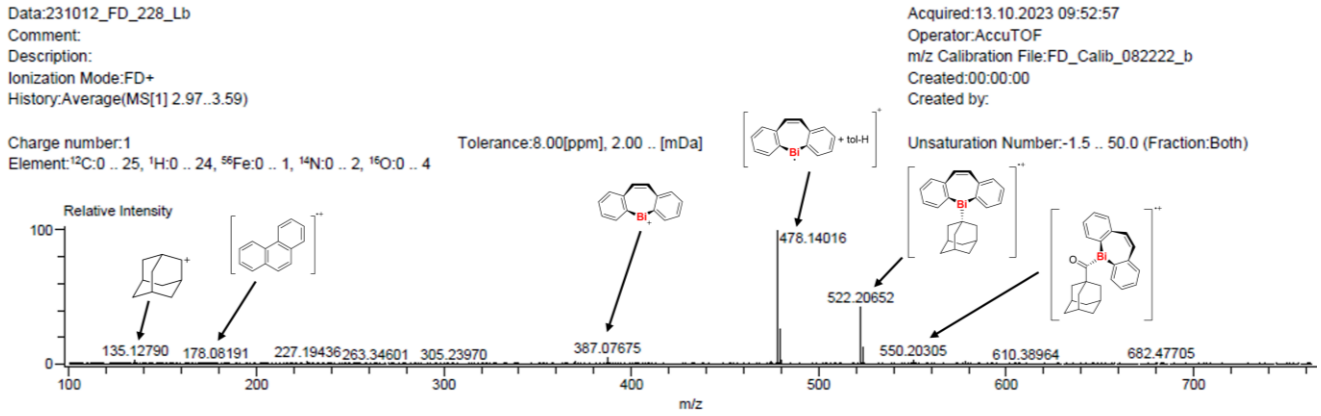

Figure S35: Experimental LIFDI-MS spectrum of compound **9** (positive mode) and identified fragments.

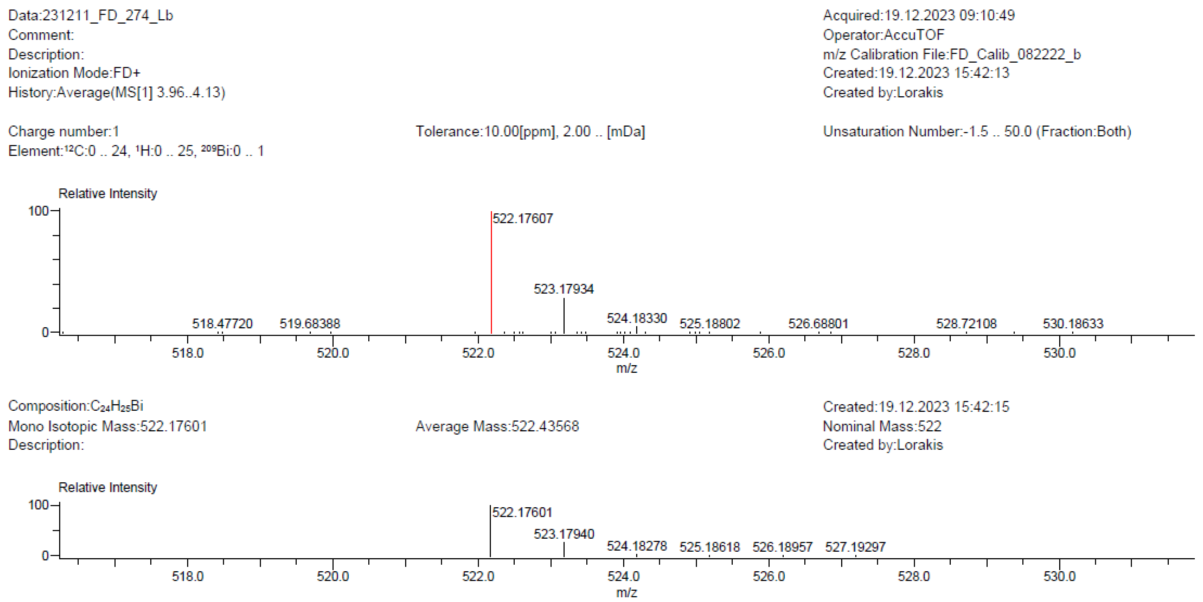

Mass	Intensity	Relative Intensity	Calc. Mass	Mass Difference [mDa]	Mass Difference [ppm]	Possible Formula	Unsaturation Number
522.17607	72718.68	100.00	522.17601	0.06	0.11	$^{12}C_{24}{}^{1}H_{25}{}^{209}Bi_1$	13.0

Figure S36: Experimental LIFDI-MS spectrum of compound **11** (positive mode, top) and simulated isotope pattern for the cation [**11**]$^+$ (bottom).

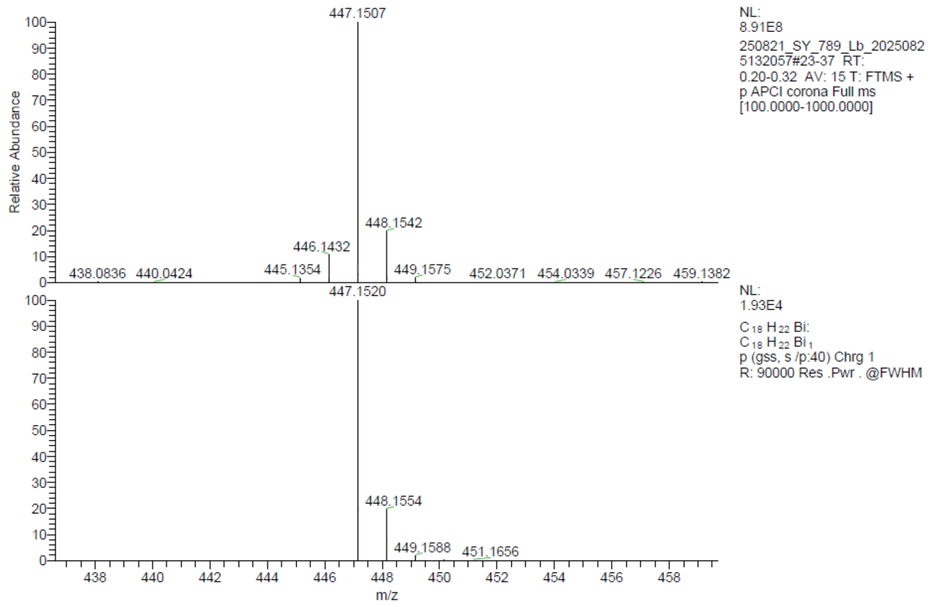

Figure S37: Experimental APCI-MS spectrum of compound **12** (positive mode, top) and simulated isotope pattern for the cation [Mes_2Bi]$^+$ (bottom).

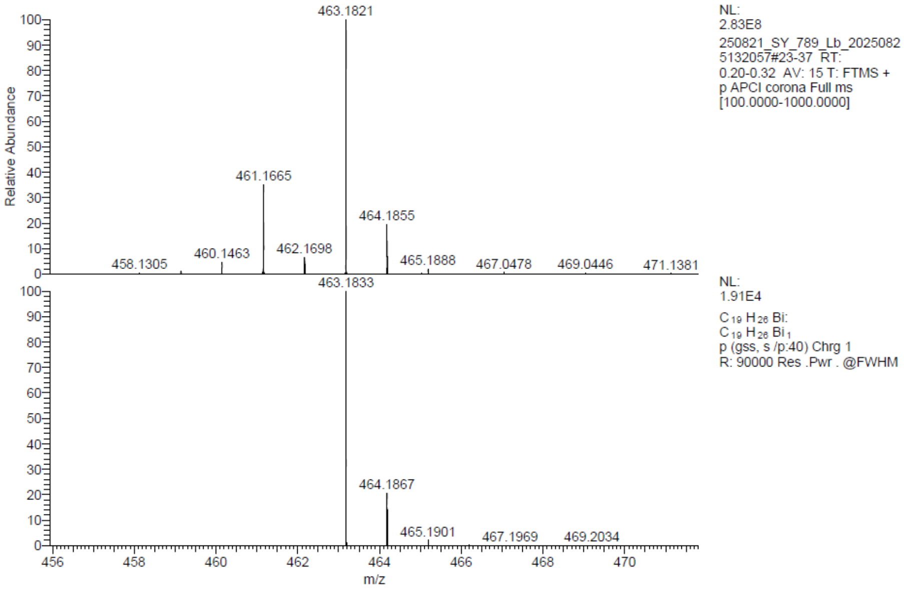

Figure S38: Experimental APCI-MS spectrum of compound **12** (positive mode, top) and simulated isotope pattern for the cation $[MesAdBi]^{+}$ (bottom).

Quantum chemical calculations

All calculations were carried out with the Amsterdam Density Functional (ADF) program using dispersion-corrected density functional theory at the ZORA-BLYP-D3(BJ)/TZP level of theory. The effect of solvation in THF was simulated by means of the Conductor like Screening Model (COSMO) of solvation as implemented in ADF. UV-Vis spectra has been computed with TD-DFT at the same level of theory.[118–127]

The through-space interaction was analyzed within the framework of quantitative Kohn-Sham molecular orbital theory in combination with a quantitative energy decomposition analysis (EDA) in the gas phase. The interaction energy ΔE_{int} was decomposed into the classical electrostatic attraction ΔV_{elstat}, Pauli repulsion ΔE_{Pauli} between occupied orbitals, stabilizing orbital interactions ΔE_{oi}, and dispersion ΔE_{disp}.[128–131] Cartesian coordinates and ADF electronic energies of systems under analysis are provided in a separate Supplementary Information file.

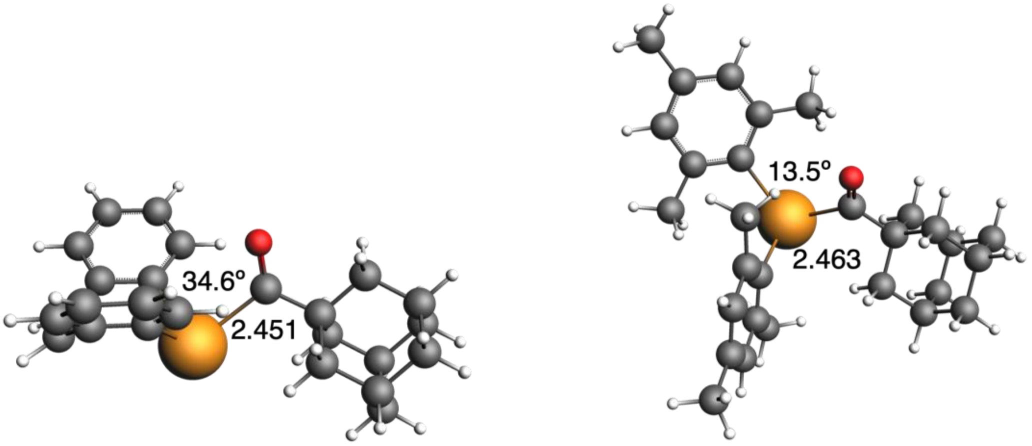

9 **10**

Figure S39: Geometry-optimized molecular structures of **9** and **10** under analysis. Bi–CO bond length (in Å) and C–Bi–C–O dihedral angles (in degrees) are enclosed. Computed at ZORA-BLYP-D3(BJ)/TZ2P in THF.

Energy decomposition analyses of the Bi···acyl interaction in compounds **9** and **10**, both homolytically and heterolytically, have been performed (Table S7 and Table S8). Based on the transfer of charge between the two fragments, the homolytic bond cleavage appears to be more realistic, as both fragments remain almost neutral. In this case its strongly pronounced covalent character is noted, as both electrostatic and orbital interactions present similar values, i.e. 45-46% of the attractive interactions in both cases. Differences between systems **9** and **10** are very small, with an almost equivalent interaction energy (–46.0 and –46.6 kcal mol^{-1} for **9** and **10**, respectively). Such similarity is also observed in the attractive electrostatic and orbital interactions. For instance, compound **10** presents slightly stronger electrostatic and orbital interactions, which is compensated by its more repulsive Pauli term. The latter is also supported by the Voronoi deformation density (VDD) charges of the fragments (Figure S40) and the data of the involved SOMOs (Figure S41).

Table S7: Energy decomposition analysis (in kcal mol^{-1}) of the homolytic breaking of the Bi···acyl interaction in compounds **9** and **10**. Computed at ZORA-BLYP-D3(BJ)/TZ2P in vacuo.

	ΔE_{Pauli}	ΔV_{elstat}	ΔE_{oi}	ΔE_{disp}	ΔE_{int}
**9**	119.8	-76.3	-76.5	-12.9	-46.0
Contribution to attractive interactions [%]		46	46	8	
**10**	125.1	-78.2	-77.1	-16.3	-46.6
Contribution to attractive interactions [%]		46	45	9	

Table S8: Energy decomposition analysis (in kcal mol^{-1}) of the heterolytic breaking of the Bi···acyl interaction in compounds **9** and **10**. Computed at ZORA-BLYP-D3(BJ)/TZ2P in vacuo.

	ΔE_{Pauli}	ΔV_{elstat}	ΔE_{oi}	ΔE_{disp}	ΔE_{int}
**9**	163.0	-161.4	-177.9	-12.9	-189.1
**10**	158.0	-153.3	-176.8	-16.3	-188.4

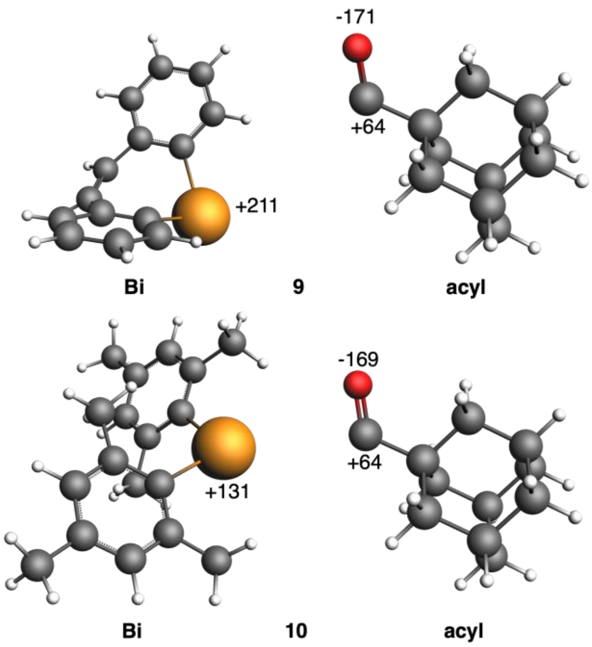

Figure S40: VDD charges (in milli-au) of frontier atoms in the analysis of the homolytic breaking of Bi···acyl interaction of systems **9** and **10**.

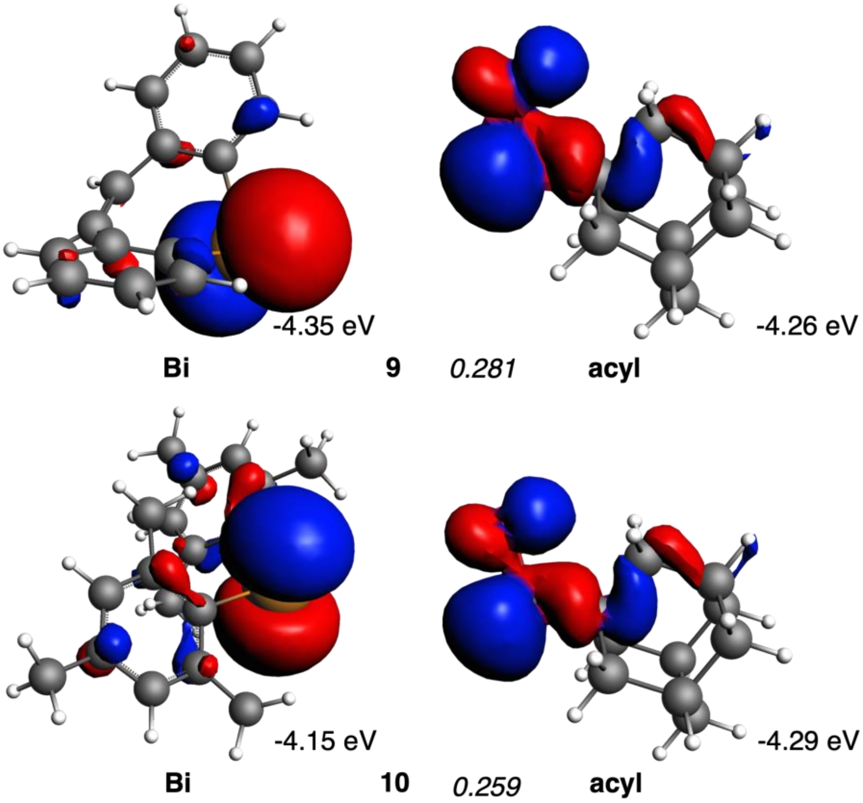

Figure S41: SOMOs orbitals in the analysis of the homolytic breaking of Bi···acyl interaction of systems **9** and **10**. Energies of the SOMOs (in eV) and their overlaps (in italics) are also enclosed. Gross Mulliken populations for the [BiR_2] fragment are 0.91, and 0.90 a.u. for **10**, and **9**, whereas those for the acyl fragment are 1.16, and 1.15 a.u., respectively. Isovalue = 0.03.

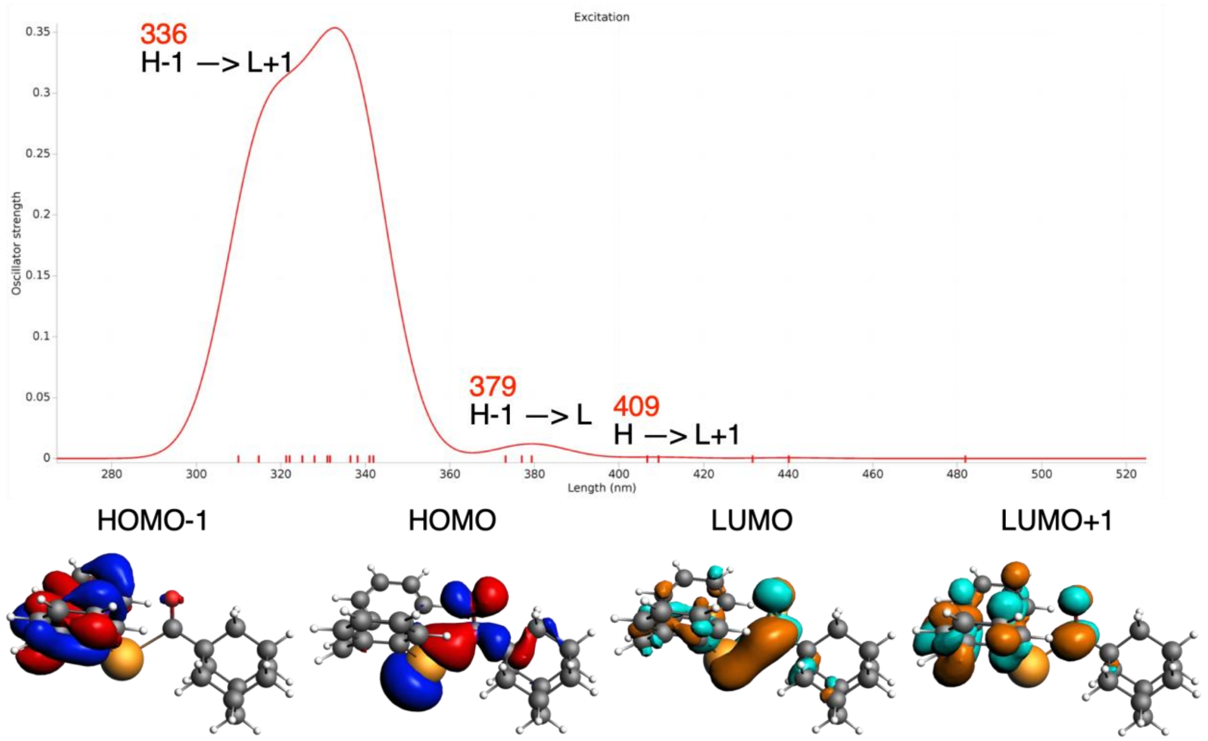

Figure S42: Calculated UV/Vis spectrum of compound **9**. Involved HOMO and LUMO orbitals are enclosed (isovalue = 0.03).

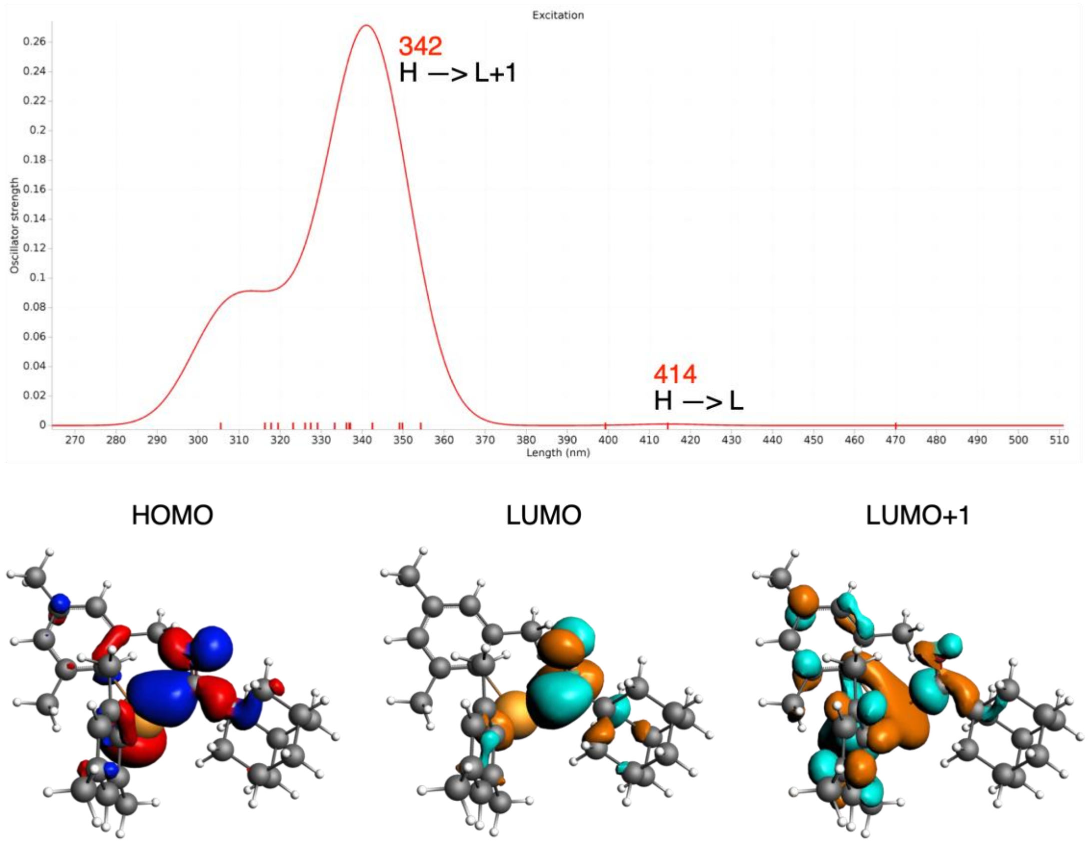

Figure S43: Calculated UV/Vis spectrum of compound **10**. Involved HOMO and LUMO orbitals are enclosed (isovalue = 0.03).

References

1. Sheldrick, G. M. A short history of SHELX. *Acta Crystallogr. A* **64,** 112–122; 10.1107/S0108767307043930 (2008).

2. Sheldrick, G. M. SHELXT - integrated space-group and crystal-structure determination. *Acta Crystallogr. A* **71,** 3–8; 10.1107/S2053273314026370 (2015).

3. Geist, F. *et al.* A Dibismuthane with Olefin Functional Groups: Towards Tridentate Hybrid Chalcogen/Olefin Ligands. *Eur. J. Inorg. Chem.,* e202300415; 10.1002/ejic.202300415 (2023).

4. Dunaj, T., Dollberg, K., Ritter, C., Dankert, F. & Hänisch, C. von. 2,6-Diisopropylphenyl-Substituted Bismuth Compounds: Synthesis, Structure, and Reactivity. *Eur. J. Inorg. Chem.* **2021,** 870–878; 10.1002/ejic.202001019 (2021).

5. Calderazzo, F., Morvillo, A., Pelizzi, G., Poli, R. & Ungari, F. Reactivity of molecules containing element-element bonds. 1. Nontransition elements. *Inorg. Chem.* **27,** 3730–3733; 10.1021/ic00294a012 (1988).

6. Rieke, R. D., Wu, T.-C. & Rieke, L. I. Highly Reactive Calcium for the Preparation of Organocalcium Reagents: 1-Adamantyl Calcium Halides and their Addition to Ketones: 1-(1-Adamantyl)cyclohexanol. *Org. Synth.* **72,** 147; 10.15227/orgsyn.072.0147 (1995).

7. Ivlev, S. I., Conrad, M. & Kraus, F. HKLF5Tools: a program for processing diffraction data of non-merohedrally twinned crystals. *Z. Kristallogr. – Cryst. Mater.* **234,** 415–418; 10.1515/zkri-2018-2147 (2019).

8. Hardman, N. J., Twamley, B. & Power, P. P. (2,6-$Mes_2H_3C_6)_2$BiH, a Stable, Molecular Hydride of a Main Group Element of the Sixth Period, and Its Conversion to the Dibismuthene (2,6-$Mes_2H_3C_6$)BiBi(2,6-$Mes_2C_6H_3$). *Angew. Chem. Int. Ed.* **39,** 2771–2773; 10.1002/1521-3773(20000804)39:15<2771::AID-ANIE2771>3.0.CO;2-7 (2000).

9. Ashe, A. J. Thermochromic Distibines and Dibismuthines. In *Advances in Organometallic Chemistry* (Elsevier1990), Vol. 30, pp. 77–97.

10. Kuczkowski, A. *et al.* Structural Characterization of Et 4 Sb 2 and Et 4 Bi 2. *Organometallics* **30,** 4730–4735; 10.1021/om2005723 (2011).

11. Mundt, O., Becker, G., Rössler, M. & Witthauer, C. Trimethylsilylverbindungen der Vb-Elemente. VII. Die Kristallstrukturen von Lithium-bis(trimethylsilyl)bismutid · DME und Tetrakis(trimethylsilyl)dibismutan sowie Bemerkungen zur Kristallstruktur des Bis(4-methoxyphenyl)ditellans. *Z. Anorg. Allg. Chem.* **506,** 42–58; 10.1002/zaac.19835061106 (1983).

12. Ashe, A. J., Kampf, J. W., Puranik, D. B. & Al-Taweel, S. M. Secondary bonding in organobismuth compounds. Comparison of the structures of 2,2',5,5'-tetramethyl-1,1'-dibismaferrocene and 2,2',5,5'-tetramethylbibismole. *Organometallics* **11,** 2743–2745; 10.1021/om00044a003 (1992).

13. Ashe, A. J. & Ludwig, E. G. A reinvestigation of Paneth's violet compound. The synthesis of tetramethyldibismuthine. *Organometallics* **1,** 1408; 10.1021/om00070a029 (1982).

14. Spence, Rupert E. v. H., Hsu, D. P. & Buchwald, S. L. Synthesis of thermochromic dibismuthines with nonthermochromic distibine analogs. *Organometallics* **11,** 3492–3493; 10.1021/om00059a003 (1992).

15. Power, P. P. Main-group elements as transition metals. *Nature* **463,** 171–177; 10.1038/nature08634 (2010).

16. Melen, R. L. Frontiers in molecular p-block chemistry: From structure to reactivity. *Science* **363,** 479–484; 10.1126/science.aau5105 (2019).

17. Martin, D., Soleilhavoup, M. & Bertrand, G. Stable singlet carbenes as mimics for transition metal centers. *Chem. Sci.* **2,** 389–399; 10.1039/C0SC00388C (2011).

18. Légaré, M.-A., Pranckevicius, C. & Braunschweig, H. Metallomimetic Chemistry of Boron. *Chem. Rev.* **119,** 8231–8261; 10.1021/acs.chemrev.8b00561 (2019).

19. Chu, T. & Nikonov, G. I. Oxidative Addition and Reductive Elimination at Main-Group Element Centers. *Chem. Rev.* **118,** 3608–3680; 10.1021/acs.chemrev.7b00572 (2018).

20. Weetman, C. & Inoue, S. The Road Travelled: After Main-Group Elements as Transition Metals. *ChemCatChem* **10,** 4213–4228; 10.1002/cctc.201800963 (2018).

21. Liebig, J. Ueber das Verhalten des Kohlenoxyds zu Kalium. *Ann. Pharm.* **11,** 182–189; 10.1002/jlac.18340110206 (1834).

22. Fujimori, S. & Inoue, S. Carbon Monoxide in Main-Group Chemistry. *J. Am. Chem. Soc.* **144,** 2034–2050; 10.1021/jacs.1c13152 (2022).

23. Wang, T. *et al.* Steric Influence on Reactions of Benzyl Potassium Species with CO. *Chem. Asian J.* **16,** 3640–3644; 10.1002/asia.202101127 (2021).

24. Wannagat, U. & Seyffert, H. Reaction of Sodium Bistrimethylsilylamide with Carbon Monoxid and Metal Carbonyls. *Angew. Chem. Int. Ed.* **4,** 438–439; 10.1002/anie.196504383 (1965).

25. Xu, M. *et al.* Facile Synthesis of Cyanide and Isocyanides from CO. *Angew. Chem. Int. Ed.* **60,** 16965–16969; 10.1002/anie.202105909 (2021).

26. Xu, M., Qu, Z.-W., Grimme, S. & Stephan, D. W. Lithium Dicyclohexylamide in Transition-Metal-Free Fischer-Tropsch Chemistry. *J. Am. Chem. Soc.* **143,** 634–638; 10.1021/jacs.0c11482 (2021).

27. Xu, M., Wang, T., Qu, Z.-W., Grimme, S. & Stephan, D. W. Reactions of a Dilithiomethane with CO and N2 O: An Avenue to an Anionic Ketene and a Hexafunctionalized Benzene. *Angew. Chem. Int. Ed.* **60,** 25281–25285; 10.1002/anie.202111486 (2021).

28. Sprangers, W., van Swieten, A. P. & Louw, R. Carbonylation of grignard compounds in ether = HMPT mixtures; a simple, one-step synthesis of ketones RC(O)CHR2 from primary alkyl derivatives RMgX. *Tetrahedron Lett.* **15,** 3377–3378; 10.1016/S0040-4039(01)91911-2 (1974).

29. Lalrempuia, R. *et al.* Activation of CO by Hydrogenated Magnesium(I) Dimers: Sterically Controlled Formation of Ethenediolate and Cyclopropanetriolate Complexes. *J. Am. Chem. Soc.* **137,** 8944–8947; 10.1021/jacs.5b06439 (2015).

30. Anker, M. D., Hill, M. S., Lowe, J. P. & Mahon, M. F. Alkaline-Earth-Promoted CO Homologation and Reductive Catalysis. *Angew. Chem. Int. Ed.* **54,** 10009–10011; 10.1002/anie.201505851 (2015).

31. Anker, M. D. *et al.* Alkaline Earth-Centered CO Homologation, Reduction, and Amine Carbonylation. *J. Am. Chem. Soc.* **139,** 10036–10054; 10.1021/jacs.7b04926 (2017).

32. Yuvaraj, K., Douair, I., Paparo, A., Maron, L. & Jones, C. Reductive Trimerization of CO to the Deltate Dianion Using Activated Magnesium(I) Compounds. *J. Am. Chem. Soc.* **141,** 8764–8768; 10.1021/jacs.9b04085 (2019).

33. Yuvaraj, K., Douair, I., Jones, D. D. L., Maron, L. & Jones, C. Sterically controlled reductive oligomerisations of CO by activated magnesium(i) compounds: deltate vs. ethenediolate formation. *Chem. Sci.* **11,** 3516–3522; 10.1039/d0sc00836b (2020).

34. Yuvaraj, K. & Jones, C. Reductive coupling of CO with magnesium anthracene complexes: formation of magnesium enediolates. *Chem. Commun.* **57,** 9224–9227; 10.1039/d1cc03890g (2021).

35. Boutland, A. J. *et al.* Reversible Insertion of a C═C Bond into Magnesium(I) Dimers: Generation of Highly Active 1,2-Dimagnesioethane Compounds. *J. Am. Chem. Soc.* **139,** 18190–18193; 10.1021/jacs.7b11368 (2017).

36. Liu, H.-Y. *et al.* Reductive Dimerization of CO by a Na/Mg(I) Diamide. *J. Am. Chem. Soc.* **143,** 17851–17856; 10.1021/jacs.1c09467 (2021).

37. Burg, A. B. & Schlesinger, H. I. Hydrides of Boron. VII. Evidence of the Transitory Existence of Borine (BH_3): Borine Carbonyl and Borine Trimethylammine. *J. Am. Chem. Soc.* **59,** 780–787; 10.1021/ja01284a002 (1937).

38. Arrowsmith, M., Böhnke, J., Braunschweig, H. & Celik, M. A. Reactivity of a Dihydrodiborene with CO: Coordination, Insertion, Cleavage, and Spontaneous Formation of a Cyclic Alkyne. *Angew. Chem. Int. Ed.* **56,** 14287–14292; 10.1002/anie.201707907 (2017).

39. Fukazawa, A. *et al.* Reaction of pentaarylboroles with carbon monoxide: an isolable organoboron carbonyl complex. *Chem. Sci.* **3,** 1814; 10.1039/c2sc20336g (2012).

40. Braunschweig, H. *et al.* Multiple complexation of CO and related ligands to a main-group element. *Nature* **522,** 327–330; 10.1038/nature14489 (2015).

41. Dahcheh, F., Martin, D., Stephan, D. W. & Bertrand, G. Synthesis and reactivity of a CAAC-aminoborylene adduct: a hetero-allene or an organoboron isoelectronic with singlet carbenes. *Angew. Chem. Int. Ed.* **53,** 13159–13163; 10.1002/anie.201408371 (2014).

42. Rang, M. *et al.* Reduction and Rearrangement of a Boron(I) Carbonyl Complex. *Angew. Chem. Int. Ed.* **60,** 2963–2968; 10.1002/anie.202014167 (2021).

43. Paetzold, P., Redenz-Stormanns, B. & Boese, R. Boroboration of CO with Tri-*tert*-butylazadiboriridine. *Angew. Chem. Int. Ed.* **29,** 900–902; 10.1002/anie.199009001 (1990).

44. Teichmann, J., Stock, H., Pritzkow, H. & Siebert, W. Carbon Monoxide and Isonitrile Insertion into the B−B Bond of Five-Membered Cyclic Organo-1,2-diboranes. *Eur. J. Inorg. Chem.* **1998,** 459–463; 10.1002/(SICI)1099-0682(199804)1998:4<459::AID-EJIC459>3.0.CO;2-U (1998).

45. Curless, L. D., Clark, E. R., Cid, J., Del Grosso, A. & Ingleson, M. J. Complete reductive cleavage of CO facilitated by highly electrophilic borocations. *Chem. Commun.* **51,** 10903–10906; 10.1039/c5cc03504j (2015).

46. Braunschweig, H. *et al.* Metal-free binding and coupling of carbon monoxide at a boron-boron triple bond. *Nat. Chem.* **5,** 1025–1028; 10.1038/nchem.1778 (2013).

47. Stephan, D. W. The broadening reach of frustrated Lewis pair chemistry. *Science* **354**; 10.1126/science.aaf7229 (2016).

48. Dureen, M. A. & Stephan, D. W. Reactions of boron amidinates with CO_2 and CO and other small molecules. *J. Am. Chem. Soc.* **132,** 13559–13568; 10.1021/ja1064153 (2010).

49. Sajid, M. *et al.* Facile carbon monoxide reduction at intramolecular frustrated phosphane/borane Lewis pair templates. *Angew. Chem. Int. Ed.* **52,** 2243–2246; 10.1002/anie.201208750 (2013).

50. Sajid, M. *et al.* Carbonylation reactions of intramolecular vicinal frustrated phosphane/borane Lewis pairs. *J. Am. Chem. Soc.* **135,** 18567–18574; 10.1021/ja408815k (2013).

51. Sajid, M., Kehr, G., Daniliuc, C. G. & Erker, G. Formylborane formation with frustrated Lewis pair templates. *Angew. Chem. Int. Ed.* **53,** 1118–1121; 10.1002/anie.201307551 (2014).

52. Cabrera, A. R. *et al.* Synthesis of new asymmetric substituted boron amidines - reactions with CO and transfer hydrogenations of phenylacetylene. *Dalton Trans.* **44,** 19606–19614; 10.1039/c5dt01966d (2015).

53. Devillard, M., Bruin, B. de, Siegler, M. A. & van der Vlugt, J. I. Transition-Metal-Free Cleavage of CO. *Chem. Eur. J.* **23,** 13628–13632; 10.1002/chem.201703798 (2017).

54. Wang, L. *et al.* Formation of macrocyclic ring systems by carbonylation of trifunctional P/B/B frustrated Lewis pairs. *Chem. Sci.* **9,** 1544–1550; 10.1039/c7sc04394e (2018).

55. Mason, M. R., Song, B. & Kirschbaum, K. Remarkable room-temperature insertion of carbon monoxide into an aluminum-carbon bond of tri-tert-butylaluminum. *J. Am. Chem. Soc.* **126,** 11812–11813; 10.1021/ja046411n (2004).

56. Mason, M. R., Song, B., Han, Y. & Hu, X. Reaction of carbon monoxide with tri-tert-butylgallium: The first example of CO insertion into a gallium–carbon bond. *Inorg. Chim. Acta* **361,** 3332–3337; 10.1016/j.ica.2007.11.021 (2008).

57. Li, X., Ni, C., Song, H. & Cui, C. Formation of aluminacyclobutenes via carbon monoxide and isocyanide insertion. *Chem. Commun.,* 1763–1765; 10.1039/b601056c (2006).

58. Kong, R. Y. & Crimmin, M. R. Reversible insertion of CO into an aluminium-carbon bond. *Chem. Commun.* **55,** 6181–6184; 10.1039/c9cc02818h (2019).

59. Heilmann, A., Hicks, J., Vasko, P., Goicoechea, J. M. & Aldridge, S. Carbon Monoxide Activation by a Molecular Aluminium Imide: C-O Bond Cleavage and C-C Bond Formation. *Angew. Chem. Int. Ed.* **59,** 4897–4901; 10.1002/anie.201916073 (2020).

60. Hudnall, T. W. & Bielawski, C. W. An N,N'-diamidocarbene: studies in C-H insertion, reversible carbonylation, and transition-metal coordination chemistry. *J. Am. Chem. Soc.* **131,** 16039–16041; 10.1021/ja907481w (2009).

61. Hudnall, T. W., Moerdyk, J. P. & Bielawski, C. W. Ammonia N-H activation by a N,N'-diamidocarbene. *Chem. Commun.* **46,** 4288–4290; 10.1039/c0cc00638f (2010).

62. Lavallo, V., Canac, Y., Donnadieu, B., Schoeller, W. W. & Bertrand, G. CO fixation to stable acyclic and cyclic alkyl amino carbenes: stable amino ketenes with a small HOMO-LUMO gap. *Angew. Chem. Int. Ed.* **45,** 3488–3491; 10.1002/anie.200600987 (2006).

63. Siemeling, U. *et al.* N-heterocyclic carbenes which readily add ammonia, carbon monoxide and other small molecules. *Chem. Sci.* **1,** 697; 10.1039/c0sc00451k (2010).

64. Martin, D., Moore, C. E., Rheingold, A. L. & Bertrand, G. An air-stable oxyallyl radical cation. *Angew. Chem. Int. Ed.* **52,** 7014–7017; 10.1002/anie.201302841 (2013).

65. Schulz, T. *et al.* Carbonylation of the simplest persistent diaminocarbene. *Chem. Commun.* **49,** 6834–6836; 10.1039/c3cc42888e (2013).

66. Paul, U. S. D. *et al.* Cyclic (Alkyl)(Amino)Carbene Complexes of Rhodium and Nickel and Their Steric and Electronic Parameters. *Chem. Eur. J.* **22,** 11005–11014; 10.1002/chem.201601406 (2016).

67. Paul, U. S. D. & Radius, U. Synthesis and Reactivity of Cyclic (Alkyl)(Amino)Carbene Stabilized Nickel Carbonyl Complexes. *Organometallics* **36,** 1398–1407; 10.1021/acs.organomet.7b00109 (2017).

68. Wang, X. *et al.* Room-temperature reaction of carbon monoxide with a stable diarylgermylene. *J. Am. Chem. Soc.* **131,** 6912–6913; 10.1021/ja9017286 (2009).

69. Ganesamoorthy, C. *et al.* A silicon-carbonyl complex stable at room temperature. *Nat. Chem.* **12,** 608–614; 10.1038/s41557-020-0456-x (2020).

70. Reiter, D., Holzner, R., Porzelt, A., Frisch, P. & Inoue, S. Silylated silicon-carbonyl complexes as mimics of ubiquitous transition-metal carbonyls. *Nat. Chem.* **12,** 1131–1135; 10.1038/s41557-020-00555-4 (2020).

71. Wang, Y. *et al.* Silicon-Mediated Selective Homo- and Heterocoupling of Carbon Monoxide. *J. Am. Chem. Soc.* **141,** 626–634; 10.1021/jacs.8b11899 (2019).

72. Xiong, Y., Yao, S., Szilvási, T., Ruzicka, A. & Driess, M. Homocoupling of CO and isocyanide mediated by a C,C'-bis(silylenyl)-substituted ortho-carborane. *Chem. Commun.* **56,** 747–750; 10.1039/c9cc08680c (2020).

73. Protchenko, A. V. *et al.* Reduction of Carbon Oxides by an Acyclic Silylene: Reductive Coupling of CO. *Angew. Chem. Int. Ed.* **58,** 1808–1812; 10.1002/anie.201812675 (2019).

74. Dietz, M. *et al.* A main-group metal carbonyl complex: Structure and isomerization to a carbene-stabilized tin atom. *Science* **389,** 1118–1121; 10.1126/science.ady0247 (2025).

75. Hansmann, M. M., Jazzar, R. & Bertrand, G. Singlet (Phosphino)phosphinidenes are Electrophilic. *J. Am. Chem. Soc.* **138,** 8356–8359; 10.1021/jacs.6b04232 (2016).

76. Puschmann, F. F. *et al.* Phosphination of carbon monoxide: a simple synthesis of sodium phosphaethynolate (NaOCP). *Angew. Chem. Int. Ed.* **50,** 8420–8423; 10.1002/anie.201102930 (2011).

77. Jupp, A. R. & Goicoechea, J. M. The 2-phosphaethynolate anion: a convenient synthesis and 2+2 cycloaddition chemistry. *Angew. Chem. Int. Ed.* **52,** 10064–10067; 10.1002/anie.201305235 (2013).

78. Xu, M., Jupp, A. R. & Stephan, D. W. Acyl-Phosphide Anions via an Intermediate with Carbene Character: Reactions of KPtBu2 and CO. *Angew. Chem. Int. Ed.* **58,** 3548–3552; 10.1002/anie.201814562 (2019).

79. Bos, A. Infrared spectroscopic evidence for germanium and tin carbonyls. *J. Chem. Soc., Chem. Commun.,* 26b; 10.1039/c3972000026b (1972).

80. Zhang, L., Dong, J. & Zhou, M. Matrix infrared spectra and quantum chemical calculations of the MCO^- (M=Si, Ge, Sn) anions. *J. Chem. Phys.* **113,** 8700–8705; 10.1063/1.1318226 (2000).

81. Jiang, L. & Xu, Q. Reactions of the Small Tin Clusters with Carbon Monoxide: Infrared Spectra and DFT Calculations of the Sn_nCO (n = 2–5) and $Sn_2(CO)_2$ Molecules in Solid Argon. *Bull. Chem. Soc. Jpn.* **79,** 857–863; 10.1246/bcsj.79.857 (2006).

82. Shi, X., Hou, C., Zhou, C., Song, Y. & Cheng, J. A Molecular Barium Hydrido Complex Stabilized by a Super-Bulky Hydrotris(pyrazolyl)borate Ligand. *Angew. Chem. Int. Ed.* **56,** 16650–16653; 10.1002/anie.201709344 (2017).

83. Ramler, J. *et al.* Carbon monoxide insertion at a heavy p-block element: unprecedented formation of a cationic bismuth carbamoyl. *Chem. Sci.* **10,** 4169–4176; 10.1039/c9sc00278b (2019).

84. In the context of this manuscript, it should be noted that Bi^{V} compounds with aryl ligands have been reported to react with CO (30 bar) in the presence of molecular sieves to give diaryl ketones, without bismuth carbonyl or bismuth acyl species having been detected: Zhao, F. & Wu, X.-F. The first bismuth self-mediated oxidative carbonlyative coupling reaction via Bi^{III}/Bi^{V} redox intermediates. *J. Catal.* **397**, 201–204; 10.1016/j.jcat.2021.03.029 (2021).

85. Kiefer, F. J., Kostenko, A., Holzner, R. & Inoue, S. Reversible CO Insertion into the Si=Si Double Bond Enables a Disila-Bislactone Formation via Subsequent CO_2 Addition. *J. Am. Chem. Soc.* **147,** 26663–26673; 10.1021/jacs.5c07040 (2025).

86. Liu, D. K., Brinkley, C. G. & Wrighton, M. S. Photochemistry of iron and ruthenium carbonyl complexes: evidence for light-induced loss of carbon monoxide and reductive elimination of triethylsilane from *cis-mer*-HM($SiEt_3$)$(CO)_3$(PPh_3). *Organometallics* **3,** 1449–1457; 10.1021/om00088a001 (1984).

87. Eskelinen, E. *et al.* Light-Induced Decarbonylation, Solvolysis, and Isomerization of Ru(L)$(CO)_2Cl_2$ (L = 2,2'-Bipyridine and 4,4'-Dimethyl-2,2'-bipyridine) in Acetonitrile. *Organometallics* **19,** 163–169; 10.1021/om9905613 (2000).

88. Alexander, J. J. Mechanism of photochemical decarbonylation of acetyldicarbonyl-.eta.5-cyclopentadienyliron. *J. Am. Chem. Soc.* **97,** 1729–1732; 10.1021/ja00840a018 (1975).

89. Szymańska-Buzar, T. Photochemical reactions of Group 6 metal carbonyls catalytic transformation of alkenes and alkynes. *Coord. Chem. Rev.* **159,** 205–220; 10.1016/S0010-8545(96)01291-X (1997).

90. Quinn, S. & Shaver, A. The photochemical and chemical decarbonylation of (η^5-C_5H_5)Fe$(CO)_2$ [*trans*C(O)CH=CHR], R = CH_3 and C_6H_5. *Inorg. Chim. Acta* **38,** 243–245; 10.1016/S0020-1693(00)91967-4 (1980).

91. King, R. B. & Bisnette, M. B. Preparation and decarbonylation of acyl derivatives of cyclopentadienyl metal carbonyls. *J. Organomet. Chem.* **2,** 15–37; 10.1016/S0022-328X(00)87596-8 (1964).

92. Cao, D. *et al.* Light-driven transition-metal-free direct decarbonylation of unstrained diaryl ketones via a dual C-C bond cleavage. *Nat. Commun.* **13,** 1805; 10.1038/s41467-022-29327-z (2022).

93. Chapman, O. L. & McIntosh, C. L. Photochemical decarbonylation of unsaturated lactones and carbonates. *J. Chem. Soc. D,* 383; 10.1039/c29710000383 (1971).

94. Küntzel, H., Wolf, H. & Schaffner, K. Photochemical Reactions. Part 63 [1]. The photodecarbonylation of α-aryl aldehydes. *Helv. Chim. Acta* **54,** 868–897; 10.1002/hlca.19710540312 (1971).

95. Poloukhtine, A. & Popik, V. V. Highly efficient photochemical generation of a triple bond: synthesis, properties, and photodecarbonylation of cyclopropenones. *J. Org. Chem.* **68,** 7833–7840; 10.1021/jo034869m (2003).

96. Poloukhtine, A. & Popik, V. V. Application of photochemical decarbonylation of cyclopropenones for the in situ generation of reactive enediynes. Construction of a cyclopropenone-containing enediyne precursor by using a cyclopropenone acetal building block. *J. Org. Chem.* **70,** 1297–1305; 10.1021/jo048065y (2005).

97. Singh, M. *et al.* Photochemical Decarbonylation of Oxetanone and Azetidinone: Spectroscopy, Computational Models, and Synthetic Applications. *Angew. Chem. Int. Ed.* **62,** e202215856; 10.1002/anie.202215856 (2023).

98. Sonobe, B. I., Fletcher, T. R. & Rosenfeld, R. N. Dynamics of the photochemical decarbonylation of 3-cyclopentenone. *J. Am. Chem. Soc.* **106,** 4352–4356; 10.1021/ja00328a009 (1984).

99. Braunschweig, H. *et al.* Main-Group Metallomimetics: Transition Metal-like Photolytic CO Substitution at Boron. *J. Am. Chem. Soc.* **139,** 1802–1805; 10.1021/jacs.6b13047 (2017).

100. Liu, L., Ruiz, D. A., Munz, D. & Bertrand, G. A Singlet Phosphinidene Stable at Room Temperature. *Chem* **1,** 147–153; 10.1016/j.chempr.2016.04.001 (2016).

101. Kiyooka, S., Shibuya, T., Shiota, F. & Fujiyama, R. Photochemical Decarbonylation of (α-Arylacyl)triphenylgermane. *Bull. Chem. Soc. Jpn.* **62,** 1361–1363; 10.1246/bcsj.62.1361 (1989).

102. Neshchadin, D. *et al.* Acylgermanes: photoinitiators and sources for Ge-centered radicals. insights into their reactivity. *J. Am. Chem. Soc.* **135,** 17314–17321; 10.1021/ja404433u (2013).

103. Radebner, J. *et al.* Tetraacylgermanes: Highly Efficient Photoinitiators for Visible-Light-Induced Free-Radical Polymerization. *Angew. Chem. Int. Ed.* **56,** 3103–3107; 10.1002/anie.201611686 (2017).

104. Frühwirt, P. *et al.* The Chemistry of Acylgermanes: Triacylgermenolates Represent Valuable Building Blocks for the Synthesis of a Variety of Germanium-Based Photoinitiators. *Inorg. Chem.* **59,** 15204–15217; 10.1021/acs.inorgchem.0c02181 (2020).

105. Mitterbauer, M. *et al.* Acylstannanes: Cleavable and Highly Reactive Photoinitiators for Radical Photopolymerization at Wavelengths above 500 nm with Excellent Photobleaching Behavior. *Angew. Chem. Int. Ed.* **57,** 12146–12150; 10.1002/anie.201804094 (2018).

106. Radebner, J. *et al.* Tetraacylstannanes as Long-Wavelength Visible-Light Photoinitiators with Intriguing Low Toxicity. *Chem. Eur. J.* **24,** 8281–8285; 10.1002/chem.201801622 (2018).

107. Jones, C., Junk, P. C., Steed, J. W., Thomas, R. C. & Williams, T. C. The interaction of 2-arsa- and 2-stiba-1,3-dionato lithium complexes with Group 8-12 metal halides. *J. Chem. Soc., Dalton Trans.,* 3219–3226; 10.1039/b105010a (2001).

108. Jones, C., Steed, J. W. & Thomas, R. C. Compounds containing λ^3,σ^2-Sb=C bonds: synthesis and structural characterisation of the first stiba-enol, Mes*C(O)Sb=C(OH)Mes* (Mes* = $C_6H_2Bu^t{}_3$-2,4,6) and a 2,3-distibabutadiene, {Mes(Me_3SiO)C=Sb}$_2$ (Mes = $C_6H_2Me_3$-2,4,6). *J. Chem. Soc., Dalton Trans.,* 1541–1542; 10.1039/a902791b (1999).

109. Weber, L. *et al.* Synthese, Struktur und Reaktivität von funktionalisierten Stibanidokomplexen des Eisens und Rutheniums [(η^5-C_5Me_5)$(CO)_2$MSbR^1R^2] (M = Fe, Ru; R^1, R^2 = $SiMe_3$, C(O)tBu, C(O)Ph, C(O)-1Ad). *Z. Anorg. Allg. Chem.* **626,** 421–429; 10.1002/(SICI)1521-3749(200002)626:2<421::AID-ZAAC421>3.0.CO;2-U (2000).

110. Black, S. J., Jones, C., Hibbs, D. E., Hursthouse, M. B. & Steed, J. W. Synthesis and structural characterisation of a novel 2,3-distibene-1,4-dione complex, [Pt(PEt_3)$_2${η^2-Bu^tC(O)Sb=SbC(O)Bu^t}]. *Chem. Commun.,* 2199–2200; 10.1039/a806712k (1998).

111. Becker, G., Becker, B., Birkhahn, M., Mundt, O. & Schmidt, R. E. Acyl- und Alkylidenarsane. VI. Vergleichende Untersuchungen zur Struktur von Bis (2,2-dimethylpropionyl) phenylarsan und -phosphan. *Z. Anorg. Allg. Chem.* **529,** 97–110; 10.1002/zaac.19855291014 (1985).

112. Müller, S. M., Schlögl, S., Wiesner, T., Haas, M. & Griesser, T. Recent Advances in Type I Photoinitiators for Visible Light Induced Photopolymerization. *ChemPhotoChem* **6,** e202200091; 10.1002/cptc.202200091 (2022).

113. Wiesner, T. & Haas, M. Do germanium-based photoinitiators have the potential to replace the well-established acylphosphine oxides? *Dalton Trans.* **50,** 12392–12398; 10.1039/D1DT02308J (2021).

114. Asthana, A. & Srivastava, R. C. Acyl derivatives of main group metals: preparation of benzoyl derivatives of some group V and VI metals and metalloids. *J. Organomet. Chem.* **366,** 281–285; 10.1016/0022-328X(89)87179-7 (1989).

115. Holownia, A., Apte, C. N. & Yudin, A. K. Acyl metalloids: conformity and deviation from carbonyl reactivity. *Chem. Sci.* **12,** 5346–5360; 10.1039/d1sc00077b (2021).

116. Ikemura, K. & Endo, T. A review of the development of radical photopolymerization initiators used for designing light-curing dental adhesives and resin composites. *Dent. Mater. J.* **29,** 481–501; 10.4012/dmj.2009-137 (2010).

117. Green, W. A. *Industrial photoinitiators. A technical guide* (CRC Press, Boca Raton, Fla., 2010).

118. te Velde, G. *et al.* Chemistry with ADF. *J. Comput. Chem.* **22,** 931–967; 10.1002/jcc.1056 (2001).

119. https://www.scm.com/ ,

120. Becke, A. D. Density-functional exchange-energy approximation with correct asymptotic behavior. *Phys. Rev. A* **38,** 3098–3100; 10.1103/physreva.38.3098 (1988).

121. Grimme, S., Antony, J., Ehrlich, S. & Krieg, H. A consistent and accurate ab initio parametrization of density functional dispersion correction (DFT-D) for the 94 elements H-Pu. *J. Chem. Phys.* **132,** 154104; 10.1063/1.3382344 (2010).

122. Grimme, S., Ehrlich, S. & Goerigk, L. Effect of the damping function in dispersion corrected density functional theory. *J. Comput. Chem.* **32,** 1456–1465; 10.1002/jcc.21759 (2011).

123. Johnson, B. G., Gill, P. M. W. & Pople, J. A. The performance of a family of density functional methods. *J. Chem. Phys.* **98,** 5612–5626; 10.1063/1.464906 (1993).

124. Lee, C., Yang, W. & Parr, R. G. Development of the Colle-Salvetti correlation-energy formula into a functional of the electron density. *Phys. Rev. B* **37,** 785–789; 10.1103/physrevb.37.785 (1988).

125. Russo, T. V., Martin, R. L. & Hay, P. J. Density functional calculations on first-row transition metals. *J. Chem. Phys.* **101,** 7729–7737; 10.1063/1.468265 (1994).

126. van Lenthe, E., Baerends, E. J. & Snijders, J. G. Relativistic total energy using regular approximations. *J. Chem. Phys.* **101,** 9783–9792; 10.1063/1.467943 (1994).

127. van Lenthe, E., van Leeuwen, R., Baerends, E. J. & Snijders, J. G. Relativistic regular two-component Hamiltonians. *Int. J. Quantum Chem.* **57,** 281–293; 10.1002/(SICI)1097-461X(1996)57:3<281::AID-QUA2>3.0.CO;2-U (1996).

128. Bickelhaupt, F. M. & Baerends, E. J. Kohn-Sham Density Functional Theory: Predicting and Understanding Chemistry. In *Reviews in Computational Chemistry,* edited by K. B. Lipkowitz & D. B. Boyd (Wiley1993), Vol. 15, pp. 1–86.

129. Bickelhaupt, F. M. & Houk, K. N. Analyzing Reaction Rates with the Distortion/Interaction-Activation Strain Model. *Angew. Chem. Int. Ed.* **56,** 10070–10086; 10.1002/anie.201701486 (2017).

130. Vermeeren, P., Hamlin, T. A. & Bickelhaupt, F. M. Chemical reactivity from an activation strain perspective. *Chem. Commun.* **57,** 5880–5896; 10.1039/d1cc02042k (2021).

131. Vermeeren, P., van der Lubbe, S. C. C., Fonseca Guerra, C., Bickelhaupt, F. M. & Hamlin, T. A. Understanding chemical reactivity using the activation strain model. *Nat. Protoc.* **15,** 649–667; 10.1038/s41596-019-0265-0 (2020).
